# Statistical Lyapunov Theory Based on Bifurcation Analysis of Energy Cascade in Isotropic Homogeneous Turbulence: A Physical–Mathematical Review

**DOI:** 10.3390/e21050520

**Published:** 2019-05-23

**Authors:** Nicola de Divitiis

**Affiliations:** Department of Mechanical and Aerospace Engineering, “La Sapienza” University, Via Eudossiana, 18, 00184 Rome, Italy; n.dedivitiis@gmail.com or

**Keywords:** energy cascade, bifurcations, Lyapunov theory

## Abstract

This work presents a review of previous articles dealing with an original turbulence theory proposed by the author and provides new theoretical insights into some related issues. The new theoretical procedures and methodological approaches confirm and corroborate the previous results. These articles study the regime of homogeneous isotropic turbulence for incompressible fluids and propose theoretical approaches based on a specific Lyapunov theory for determining the closures of the von Kármán–Howarth and Corrsin equations and the statistics of velocity and temperature difference. While numerous works are present in the literature which concern the closures of the autocorrelation equations in the Fourier domain (i.e., Lin equation closure), few articles deal with the closures of the autocorrelation equations in the physical space. These latter, being based on the eddy–viscosity concept, describe diffusive closure models. On the other hand, the proposed Lyapunov theory leads to nondiffusive closures based on the property that, in turbulence, contiguous fluid particles trajectories continuously diverge. Therefore, the main motivation of this review is to present a theoretical formulation which does not adopt the eddy–viscosity paradigm and summarizes the results of the previous works. Next, this analysis assumes that the current fluid placements, together with velocity and temperature fields, are fluid state variables. This leads to the closures of the autocorrelation equations and helps to interpret the mechanism of energy cascade as due to the continuous divergence of the contiguous trajectories. Furthermore, novel theoretical issues are here presented among which we can mention the following ones. The bifurcation rate of the velocity gradient, calculated along fluid particles trajectories, is shown to be much larger than the corresponding maximal Lyapunov exponent. On that basis, an interpretation of the energy cascade phenomenon is given and the statistics of finite time Lyapunov exponent of the velocity gradient is shown to be represented by normal distribution functions. Next, the self–similarity produced by the proposed closures is analyzed and a proper bifurcation analysis of the closed von Kármán–Howarth equation is performed. This latter investigates the route from developed turbulence toward the non–chaotic regimes, leading to an estimate of the critical Taylor scale Reynolds number. A proper statistical decomposition based on extended distribution functions and on the Navier–Stokes equations is presented, which leads to the statistics of velocity and temperature difference.

## 1. Introduction

This article presents a review of previous works of the author regarding an original Lyapunov analysis of the developed turbulence which leads to the closures of the von Kármán–Howarth and Corrsin equations and to the statistics of both velocity and temperature difference [1,2,3,4,5,6,7]. This theory studies the fully developed homogeneous isotropic turbulence through the bifurcations of the incompressible Navier–Stokes equations using a specific statistical Lyapunov analysis of the fluid kinematic field. In addition, now it is introduced the energy cascade interpretation and explained some of the mathematical properties of the proposed closures. This work is organized into two parts. One is the reasoned review of previous results but with new demonstrations and theoretical procedures. The other one, presented in sections marked with asterisk symbol “*”, concerns new theoretical issues of the proposed turbulence theory.

Although numerous articles were written which concern the closures of the Lin equation in the Fourier domain [8,9,10,11,12,13,14,15,16], few works address the closures of the autocorrelation equations in the physical space. These last ones, being based on the eddy–viscosity concept, describe diffusive closure models. Unlike the latter, the proposed Lyapunov theory provides nondiffusive closures in the physical space based on the property that, in developed turbulence, contiguous fluid particles trajectories continuously diverge. Thus, the main purpose of this review is to summarize the results of the previous works based on a theory which does not use the eddy–viscosity paradigm and to give new theoretical insights into some related issues.

The homogeneous isotropic turbulence is an ideal flow regime characterized by the energy cascade phenomenon where the diverse parts of fluid exhibit the same statistics and isotropy. On the other hand, the turbulent flows occurring in nature and in the various fields of engineering are generally much more complex than homogeneous isotropic turbulence. In such flows, spatial variations of average velocity and of other statistical flow properties can happen causing very complex simultaneous effects that add to the turbulent energy cascade and interact with the latter in a nontrivial fashion. Hence, the study of the energy cascade separately from the other phenomena requires the analysis of isotropic homogenous turbulence.

The von Kármán–Howarth and Corrsin equations are the evolution equations of longitudinal velocity and temperature correlations in homogeneous isotropic turbulence, respectively. Both the equations, being unclosed, need the adoption of proper closures [17,18,19,20]. In detail, the von Kármán–Howarth equation includes *K*, the term due to the inertia forces and directly related to the longitudinal triple velocity correlation *k*, which has to be properly modelled. The modeling of such term must take into account that, due to the inertia forces, *K* does not modify the kinetic energy and satisfies the detailed conservation of energy [18]. This latter states that the exchange of energy between wave–numbers is only linked to the amplitudes of such wave–numbers and of their difference [21]. Different works propose for the von Kármán–Howarth equation the diffusion approximation [22,23,24]

(1) $$\begin{array}{c} k=2\frac{D}{u}\frac{\partial f}{\partial r} \end{array}$$

where *r* and D=D(r) are separation distance and turbulent diffusion parameter, respectively and $u^{2}=\langle u_{i}u_{i}\rangle /3$ corresponds to the longitudinal velocity standard deviation. Following Equation (1), the turbulence can be viewed as a diffusivity phenomenon depending upon *r*, where *K* will include a term proportional to $\partial^{2}f/\partial r^{2}$. In the framework of Equation (1), Hasselmann [22] proposed, in 1958, a closure suggesting a link between *k* and *f* which expresses *k* in function of the momentum convected through a spherical surface. His model, which incorporates a free parameter, expresses D(r) by means of a complex expression. Thereafter, Millionshtchikov developed a closure of the form $D\left(r\right)=k_{1}ur$, where $k_{1}$ represents an empirical constant [23]. Although both the models describe two possible mechanisms of energy cascade, in general, do not satisfy some physical conditions. For instance, the Hasselmann model does not verify the continuity equation for all the initial conditions, whereas the Millionshtchikov equation gives, following Equation (1), values of velocity difference skewness in contrast with experiments and energy cascade [18]. More recently, Oberlack and Peters [24] suggested a closure where $D\left(r\right)=k_{2}ru\sqrt{1-f}$, being $k_{2}$ a constant parameter. The authors show that such closure reproduces the energy cascade and, for a proper choice of $k_{2}$, provides results in agreement with the experiments [24].

For what concerns the Corrsin equation, this exhibits *G*, the term responsible for the thermal energy cascade. This quantity, directly related to the triple velocity–temperature correlation $m^{*}$, also needs adequate modellation. As *G* depends also on the velocity correlation, the Corrsin equation requires the knowledge of *f*, thus it must be solved together to the von Kármán–Howarth equation. Different works can be found in the literature which deal with the closure of Corrsin equation. Some of them study the self-similarity of the temperature correlation in order to analyze properties and possible expressions for *G*. Such studies are supported by the idea that the simultaneous effect of energy cascade, conductivity and viscosity, makes the temperature correlation similar in the time. This question was theoretically addressed by George (see [25,26] and references therein) which showed that the decaying isotropic turbulence reaches the self–similarity, while the temperature correlation is scaled by the Taylor microscale whose current value depends on the initial condition. More recently, Antonia et al. [27] studied the temperature structure functions in decaying homogeneous isotropic turbulence and found that the standard deviation of the temperature, as well as the turbulent kinetic energy, follows approximately the similarity over a wide range of length scales. There, the authors used this approximate similarity to estimate the third–order correlations and found satisfactory agreement between measured and calculated functions. On the other hand, the temperature correlation can be obtained using proper closures of von Kármán–Howarth and Corrsin equations suitable for the energy cascade phenomenon. On this argument, several articles has been written. For instance, Baev and Chernykh [28] (and references therein) analyzed velocity and temperature correlations by means of a closure model based on the gradient hypothesis which relates pair longitudinal second and third order correlations, by means of empirical coefficients.

Although other works regarding the von Kármán–Howarth equation were written [29,30,31,32,33], to the author’s knowledge a physical–mathematical analysis based on basic principles which provides analytical closures of von Kármán–Howarth and Corrsin equations has not received due attention. Therefore, the aim of the this work is to present a review of the Lyapunov analysis presented in [1,2,3,4,5,6,7] and new theoretical insights into some related issues.

In the present formulation, based on the Navier–Stokes bifurcations, the current fluid placements, together with velocity and temperature fields, are considered to be fluid state variables. This leads to the closures of the autocorrelation equations and helps to interpret the mechanism of energy cascade as due to the continuous divergence of the contiguous trajectories.

In line with Ref. [3], the present work first addresses the problem for defining the bifurcations for incompressible Navier–Stokes equations, considering that these latter can be reduced to an opportune symbolic form of operators for which the classical bifurcation theory of differential equations can be applied [34]. In such framework, this analysis remarks that a single Navier–Stokes bifurcation will generate a doubling of the velocity field and of all its several properties, with particular reference to the characteristic length scales. If on one side the lengths are doubled due to bifurcations, on the other hand the characteristic scale for homogeneous flows in infinite domains is not defined. Hence, the problem to define the characteristic length—and therefore the flow Reynolds number—in such situation is also discussed. Such characteristic scale is here defined in terms of spatial variations of initial or current velocity field in such a way that, in fully developed homogeneous isotropic turbulence, this length coincides with the Taylor microscale. As far as the characteristic velocity is concerned, this is also defined in terms of velocity field so that, in developed turbulence, identifies the velocity standard deviation.

The trajectories bifurcations in the phase space of the velocity field are here formally dealt with using a proper Volterra integral formulation of the Navier–Stokes equations, whereas the turbulence transition is qualitatively analyzed through general properties of the bifurcations and of the route toward the fully developed chaos. This background, regarding the general bifurcations properties and the route toward the chaos, will be useful for this analysis.

The adopted statistical Lyapunov theory shows how the fluid relative kinematics can be much more rapid than velocity and temperature fields in developed turbulence, so that fluid strain and velocity fields are statistically independent with each other. Moreover, in addition to References [1,2,3,4,5,6,7], this analysis introduces the bifurcation rate of the velocity gradient, a quantity providing the frequency at which the velocity gradient determinant vanishes along fluid particles trajectories. The bifurcation rate, in fully developed turbulence, is shown to be much greater than the maximal Lyapunov exponent of the velocity gradient. This explains the energy cascade through the relation between material vorticity, Lyapunov vectors and bifurcation rate using the Lyapunov theory. In detail, the energy cascade can be viewed as a continuous and intensive stretching and folding process of fluid particles which involves smaller and smaller length scales during the fluid motion, where the folding frequency equals the bifurcation rate.

Next, the statistics of the Lyapunov exponents is reviewed. In agreement with Reference [6], we show that the local Lyapunov exponents are uniformely unsymmetrically distributed in their interval of variation. Unlike Reference [6] which uses the criterion of maximum entropy associated with the fluid particles placements, the isotropy and homogeneity hypotheses are here adopted. A further result with respect to the previous issues pertains the finite time Lyapunov exponents statistics: through the bifurcation analysis and the central limit theorem, we show that the finite time Lyapunov exponent tends to a fluctuating variable distributed following a normal distribution function.

Thereafter, the closure formulas of von Kármán–Howarth and Corrsin equations are derived through the Liouville equation and finite scale Lyapunov exponent statistics. These closures do not correspond to a diffusive model, being the result of the trajectories’ divergence in the continuum fluid. Such formulas coincide with those just obtained in References [1,4,5] where it is shown that such closures adequately describe the energy cascade phenomenon, reproducing, negative skewness of velocity difference, the Kolmogorov law and temperature spectra in line with the theoretical argumentation of Kolmogorov, Obukhov–Corrsin and Batchelor [35,36,37], with experimental results [38,39] and with numerical data [40,41]. These closures are here achieved by using different mathematical procedures with respect to the other articles [1,4,5]. While the previous works derive such closures studying the local fluid act of motion in the finite scale Lyapunov basis [1,4] and adopting maximum and average finite scale Lyapunov exponents [5], here these closures are obtained by means of the local finite scale Lyapunov exponents PDF, showing that the assumptions of References [1,4,5] agree with this analysis, corroborating the previous results. Some of the properties of the proposed closures are then studied, with particular reference to the evolution times of the developed correlations and their self–similarity. In detail, as new result with respect the previous articles, this analysis shows that the proposed closures generate correlations self–similarity in proper ranges of separation distance, which is directly linked to the particles trajectories divergence.

Furthermore, a novel bifurcation analysis of the closed von Kármán–Howarth equation is proposed, which considers the route starting from the fully developed turbulence toward the non–chaotic regimes. This extends the discussion of the previous works and represents an alternative point of view for studying the turbulent transition. According to this analysis, the closed von Kármán–Howarth equation is decomposed in several ordinary differential equations through the Taylor series expansion of the longitudinal velocity correlation. This procedure, which also accounts for the aforementioned self–similarity, leads to estimating the Taylor scale Reynolds number at the transition. This latter is found to be 10, a value in good agreement with several experiments which give values around 10, and in particular with the bifurcations analysis of the energy cascade of Reference [3], which provides a critical Reynolds number of 10.13 if the route toward the turbulence follows the Feigenbaum scenario [42,43].

Finally, the statistics of velocity and temperature difference, of paramount importance for estimating the energy cascade, is reviewed. While References [1,2,4,7] determine such statistics through a concise heuristic method, this analysis uses a specific statistical decomposition of velocity and temperature which adopts appropriate stochastic variables related to the Navier–Stokes bifurcations. The novelty of the present approach with respect to the previous articles is that the random variables of such decomposition are opportunely chosen to reproduce the Navier–Stokes bifurcation effects and the isotropy: these are highly nonsymmetrically distributed stochastic variables following opportune extended distribution functions which can assume negative values. Such decomposition, able to reproduce negative skewness of longitudinal velocity difference, provides a statistics of both velocity and temperature difference in agreement with theoretical and experimental data known from the literature [44,45,46,47,48]. Here, in addition to References [1,2,4,7], a detailed mathematical analysis is presented which concerns the statistical properties of the aforementioned extended distribution functions in relation to the Navier–Stokes bifurcations.

In brief, the original contributions of the present work can be summarized as:

(i) The bifurcation rate associated with the velocity gradient is shown to be much larger than the maximal Lyapunov exponent of the velocity gradient.
(ii) As the consequence of (i), the energy cascade can be viewed as a succession of stretching and folding of fluid particles which involves smaller and smaller length scales, where the particle folding happens at the frequency of the bifurcation rate.
(iii) As the consequence of (i), the central limit theorem provides reasonable argumentation that the finite time Lyapunov exponent is distributed following a gaussian distribution function.
(iv) The proposed closures generate correlations self–similarity in proper ranges of variation of the separation distance which is directly caused by the continuous fluid particles trajectories divergence.
(v) A specific bifurcation analysis of the closed von Kármán–Howarth equation is proposed which allows to estimate the critical Taylor scale Reynolds number in isotropic turbulence.
(vi) A statistical decomposition of velocity and temperature is presented which is based on stochastic variables distributed following extended distribution functions. Such decomposition leads to the statistics of velocity and temperature difference, where the intermittency of these latter increases as Reynolds number and Péclet number rise.

## 2. Background

In the framework of the link between bifurcations and turbulence, this section deals with some of the fundamental elements of the Navier–Stokes equations and heat equation, useful for the present analysis. In particular, we will address the problem of defining an adequate bifurcation analysis for the Navier–Stokes equations and will analyze the meaning of the characteristic length scales when a homogeneous flow is in an infinite domain. All the considerations regarding the fluid temperature can also be applied to any passive scalar that exhibits diffusivity. A statistically homogeneous and isotropic flow with null average velocity is considered.

In order to formulate the bifurcation analysis, we start from the Navier–Stokes equations and the temperature equation

(2) $$\begin{array}{c} \nabla_{x}\cdot u=0,\\ \frac{\partial u}{\partial t}=-\nabla_{x}u\;u-\frac{\nabla_{x}p}{\rho }+\nu \nabla_{x}^{2}u \end{array}$$

(3) $$\frac{\partial \vartheta }{\partial t}=-u\cdot \nabla_{x}\vartheta +\chi \nabla_{x}^{2}\vartheta$$

where u = u(t,x), *p* = p(t,x) and ϑ = ϑ(t,x) are velocity, pressure and temperature fields, ν and χ = $k\rho /C_{p}$ are fluid kinematic viscosity and thermal diffusivity, being ρ = const, *k* and $C_{p}$ density, fluid thermal conductivity and specific heat at constant pressure, respectively. In this study ν and χ are supposed to be independent from the temperature, thus Equation (2) is autonomous with respect to Equation (3), whereas Equation (3) will depend on Equation (2).

To define the bifurcations of Equations (2) and (3), such equations are first expressed in the symbolic form of operators. To this end, in the momentum Navier–Stokes equations, the pressure field is eliminated by means of the continuity equation, thus Equations (2) and (3) are formally written as

(4) $$\dot{u}=N\left(u;\nu \right),$$

(5) $$\dot{\vartheta }=M\left(u,\vartheta ;\chi \right)$$

in which N is a nonlinear quadratic operator incorporating $-u\cdot \nabla_{x}u$, $-\nu \nabla_{x}^{2}u$ and the integral nonlinear operator which expresses the pressure gradient as a functional of the velocity field, being

(6) $$p\left(t,x\right)=\frac{\rho }{4\pi }\int \frac{\partial^{2}u_{i}^{\prime }u_{j}^{\prime }}{\partial x_{i}^{\prime }\partial x_{j}^{\prime }}\;\frac{dV(x^{\prime })}{|x^{\prime }-x|}$$

Therefore, *p* provides nonlocal effects of the velocity field [49] and the Navier–Stokes equations are reduced to be an integro–differential equation formally expressed by Equation (4). For what concerns Equation (5), it is the evolution equation of ϑ, where M is a linear operator of ϑ. Accordingly, transition and turbulence are caused by the bifurcations of Equation (4), where $\nu^{-1}$ plays the role of the control parameter. At this stage of the analysis, it is worth to remark the following two items: (a) there is no explicit methods of bifurcation analysis for integro–differential equations such as Equation (4). (b) since the flow is statistically homogeneous in an infinite domain, characteristic scales of the problem are not defined.

The item (a) can be solved according to the analysis method proposed by Ruelle and Takens in Reference [34]: it is supposed that the infinite dimensional space of velocity field $\left\{u\right\}$ can be replaced by a finite–dimensional manifold, then Equation (4) can be reduced to be the equation of the kind studied by Ruelle and Takens in Reference [34]. Therefore, the classical bifurcation theory of ordinary differential equations [34,43,50] can be formally applied to Equation (4) and the present analysis can be considered valid within the limits of the formulation proposed in Reference [34].

For what concerns the characteristic length, a homogeneous flow in infinite domain is free from boundary conditions, thus the characteristic scale, being not defined, is here chosen in function of the spatial variations of the current velocity field. Thus, for all flow regimes in infinite regions, (i.e., non–chaotic, turbulent and transition flows), characteristic length and velocity, *L* and *U* respectively, are here chosen in terms of volume integrals of u in the following manner

(7) $$\begin{array}{l} U^{2}=\lim_{V\to \infty }\frac{1}{V}\int_{V}u\left(t,x\right)\cdot u\left(t,x\right)dV\left(x\right),\\ G^{2}=\lim_{V\to \infty }\frac{1}{V}\int_{V}\nabla_{x}u:\nabla_{x}u\;dV\left(x\right),\\ L^{2}=c\;\frac{U^{2}}{G^{2}} \end{array}$$

where V is the fluid domain volume, “:” denotes the Frobenius inner product and *c* = O(1) is a dimensionless constant which will be properly chosen. The flow Reynolds number is then defined in terms of *U* and *L* as

(8) $$Re=\frac{UL}{\nu }.$$

Equation (8) provides an extension of the Taylor scale Reynolds number which applies for every flow regime. In particular, such definition holds also for non turbulent flows, where *U* and *L*, although not velocity standard deviation and statistical correlation scale, provide a generalization of the latter. In fully developed homogeneous turbulence, the volume integrals appearing in Equation (7) equal statistical averages calculated over the velocity field ensemble, such as velocity standard deviation and dissipation rate. Accordingly, in isotropic homogeneous turbulence, *L* and *U* identify, respectively, the Taylor scale $\lambda_{T}$ and standard deviation *u* of one of the velocity components and Re=UL/ν coincides with the Taylor scale Reynolds number $R_{T}$. Such definitions (7) extend the concept of velocity variance and statistical correlation scale and will be used for the bifurcation analysis proposed in this work.

## 3. Navier–Stokes Bifurcations

Before introducing the bifurcations analysis of the Navier–Stokes equations in the operatorial form (4), it is worth remarking that a given point in the space of velocity fields set $\bar{u}\in \left\{u\right\}$—or temperature field $\bar{\vartheta }\in \left\{\vartheta \right\}$—corresponds to a spatial distribution including all its characteristics, in particular the length scales associated with $\bar{u}$.

The bifurcations of Equation (4) happen when the Jacobian $\nabla_{u}N$ exhibits at least an eigenvalue with zero real part (NS–bifurcations), and this occurs when

(9) $$\det(\nabla_{u}N)=0.$$

Such bifurcations are responsible for multiple velocity fields $\hat{u}$ which provides the same field $\dot{u}$. In fact, during the fluid motion, multiple solutions $\hat{u}$ and $\hat{\vartheta }$ can be determined, at each instant, through inversion of Equation (4)

(10) $$\begin{array}{c} \dot{u}=N\left(u;\nu \right)\\ \hat{u}=N^{-1}\left(\dot{u};\nu \right),\\ \hat{\vartheta }=M^{-1}\left(\dot{\vartheta },\hat{u};\chi \right) \end{array}$$

In the framework of the trajectories bifurcations in the phase space, the fluid motion can be expressed by means of Equation (4) and initial conditions u(0) and ϑ(0), using the following Volterra integral formulation

(11) $$\begin{array}{c} u\left(t\right)-u\left(0\right)-\int_{0}^{t}N\left(u\left(\tau \right);\nu \right)\;d\tau \equiv N\left(u;\nu \right)=0,\\ \vartheta \left(t\right)-\vartheta \left(0\right)-\int_{0}^{t}M\left(u\left(\tau \right),\vartheta \left(\tau \right);\chi \right)\;d\tau \equiv M\left(u,\vartheta ;\chi \right)=0 \end{array}$$

where N and M are proper operators such that

(12) $$\begin{array}{c} \begin{array}{c} N:\left\{u\right\}\to N\left(\left\{u\right\}\right),\\ M:\left\{u\right\}\times \left\{\vartheta \right\}\to M\left(\left\{u\right\}\times \left\{\vartheta \right\}\right) \end{array} \end{array}$$

Specifically, N is a nonlinear operator of u, where the image $N(\left\{u\right\})$ has the same structure of $\left\{u\right\}$, whereas M is linear with respect to ϑ and the image $M(\left\{u\right\}\times \left\{\vartheta \right\})$ is isomorphic with $\left\{\vartheta \right\}$. Thus, N and M admit in general the following jacobians

(13) $$\begin{array}{c} \begin{array}{c} \nabla_{u}N,\\ \nabla_{\vartheta }M \end{array} \end{array}$$

According to Equation (11), a trajectory bifurcation happens when $\nabla_{u}N$ is singular, that is when

(14) $$\det\left(\nabla_{u}N\right)=0$$

and the multiple solutions of Equation (11), say $\hat{u}$ and $\hat{\vartheta }$, are given in terms of u and ϑ through

(15) $$\begin{array}{c} \begin{array}{c} N\left(\hat{u};\nu \right)=N\left(u;\nu \right)=0,\\ M\left(\hat{u},\hat{\vartheta };\chi \right)=M\left(u,\vartheta ;\chi \right)=0 \end{array} \end{array}$$

using the implicit functions theorem. Therefore, if velocity and temperature fields are supposed to be known for $\nu =\nu_{0}$, the fields calculated for $\nu \neq \nu_{0}$ are formally expressed as

(16) $$\begin{array}{c} \begin{array}{c} \hat{u}\left(\nu \right)=u\left(\nu_{0}\right)-\int_{\nu_{0}}^{\nu }{\left(\nabla_{u}N\right)}^{-1}\frac{\partial N}{\partial \nu }\;d\nu ,\\ \hat{\vartheta }\left(\nu \right)=\vartheta \left(\nu_{0}\right)+\int_{\nu_{0}}^{\nu }{\left(\nabla_{\vartheta }M\right)}^{-1}\left(\nabla_{u}M\right){\left(\nabla_{u}N\right)}^{-1}\frac{\partial N}{\partial \nu }\;d\nu , \end{array} \end{array}$$

## 4. Qualitative Analysis of the Route Toward the Chaos

With reference to Equation (10) or (16), when $\nu^{-1}$ is relatively small, N and N behave like linear operators and Equation (15) returns $\hat{u}\equiv u\left(t,x\right)$ as unique solution. Increasing $\nu^{-1}$, the Navier–Stokes equations encounter the first bifurcation at $\nu =\nu_{1}$, the jacobian $\nabla_{u}N$ is singular there, and thereafter Equation (15) determines different velocity fields $\hat{u}$ with the corresponding length scales. A single bifurcation causes a doubling of u, that is, a doubling of the velocity values and of the length scales. Although the route toward the chaos can be of different kinds [34,42,43,51], one common element of these latter is that the number of encountered bifurcations at the onset of the chaotic regimes is about greater than three. Hence, if $\nu^{-1}$ is quite small, the velocity field can be represented by its Fourier series of a given basic scale. The first bifurcation introduces new solutions $\hat{u}$ whose Fourier characteristic lengths are independent from the previous one. Thereafter, each bifurcation adds new independent scales, and, after the third bifurcation ($\nu^{-1}=\nu_{*}^{-1}$), the transition occurs, the several characteristic lengths and the velocity values appear to be continuously distributed and thus the velocity field is represented by the Fourier transform there. In such situations, a huge number of such solutions are unstable; u(t,x) tends to sweep the entire velocity field set and the motion is expected to be chaotic with a high level of mixing. As for $\hat{\vartheta }$, M and M are both linear operators of ϑ, thus $\hat{\vartheta }$ follows the variations of $\hat{u}$.

If $\nu^{-1}$ does not exceed its critical value, say $\nu_{*}^{-1}$, the velocity fields satisfying Equation (15) are limited in number and this corresponds to the intermediate stages of the route toward the chaos. On the contrary, when $\nu^{-1}>\nu_{*}^{-1}$, the region of developed turbulence where $\lambda_{NS}>$ 0 is observed, being $\lambda_{NS}$ the average maximal Lyapunov exponent of the Navier–Stokes equations, is formally calculated as

(17) $$\begin{array}{c} \begin{array}{c} \lambda_{NS}=\lim_{T\to \infty }\frac{1}{T}\int_{0}^{T}\frac{y\cdot \nabla_{u}Ny}{y\cdot y}\;dt,\\ \dot{y}=\nabla_{u}N\left(u;\nu \right)y,\\ \dot{u}=N\left(u;\nu \right), \end{array} \end{array}$$

and *y* is the Lyapunov vector associated with the Navier–Stokes equations. Then, $\nu_{*}^{-1}$ depends on u, and $Re_{*}$, calculated with Equation (7), can be roughly estimated as the minimum value of Re for which $\lambda_{NS}\geq$ 0.

**Figure 1:**
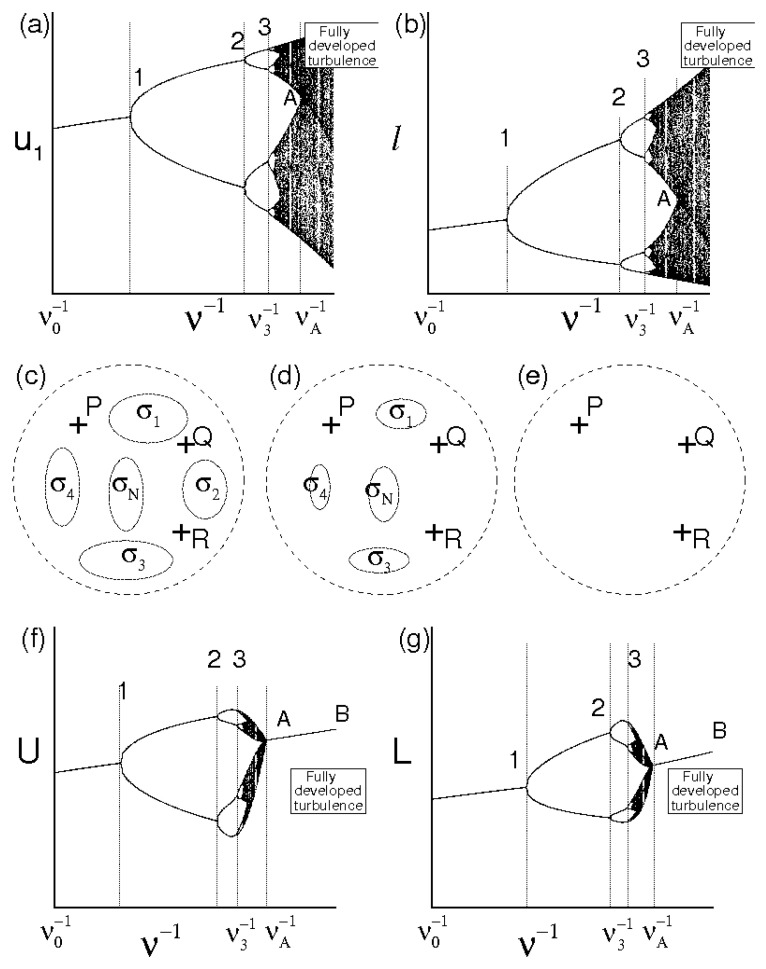
Qualitative scheme of the route toward the turbulence. (**a**,**b**): velocity and length scale in terms of kinematic viscosity. (**c**–**e**): symbolic representation of solutions in the velocity fields set. (**f**,**g**): U and L in terms of kinematic viscosity.

Figure 1 qualitatively shows the route from non–chaotic regimes toward the developed turbulence. Specifically, Figure 1a,b report two bifurcation maps at a given instant, providing the velocity component $u_{1}$ in a point of the space and one characteristic scale *ℓ* of the velocity field in function of $\nu^{-1}$. Figure 1c–e symbolically represent, for assigned values of ν, the velocity field set (points inside the dashed circle), three different solutions of the Navier–Stokes equations—say P, Q and R—and the several subsets $\sigma_{1}$, $\sigma_{2}$,… which correspond to islands that are not swept during the fluid motion. The figure also depicts *L*=$L(\nu^{-1})$ and *U*=$U(\nu^{-1})$ (Figure 1f,g), formally calculated with Equation (7). Following Equation (16), these maps are not universal, as $u_{1}$ = $u_{1}\left(\nu^{-1}\right)$, $\ell =\ell (\nu^{-1})$, *L* = $L(\nu^{-1})$ and *U* = $U(\nu^{-1})$ do not represent universal laws and their order of magnitude will depend on velocity field at $\nu_{0}^{-1}$. When $\nu^{-1}>\nu_{3}^{-1}$, the number of solutions diverges and the bifurcation tree of $u_{1}$ and *ℓ* drastically changes its structure showing tongue geometries that develop from the different bifurcations. As long as $\nu^{-1}$ does not exceed much $\nu_{3}^{-1}$, the extension of such tongues is relatively bounded, whereas the measure of the islands $\sigma_{k}$ is quite large. This means that, although $u_{1}$ and *ℓ* exhibit chaotic behavior there, these do not sweep completely their variation interval, thus Equation (4) do not behave like an ergodic dynamic system there. This corresponds to Figure 1c, where the velocity fields P, Q and R, being differently placed with respect to $\sigma_{k}$, k=1,2,… will exhibit different values of average kinetic energy and dissipation rate in V. As $\nu^{-1}$ rises, these tongues gradually increase their extension whereas the measures of $\sigma_{k}$ diminish (see Figure 1d) until reaching a situation in which the bifurcation tongues overlap with each other and the islands $\sigma_{k}$ vanish (Figure 1e). Such developed overlapping corresponds to the chaotic behavior of $u_{1}$ and *ℓ*, where these latter almost entirely describe their variation interval: Equation (4) behave like an ergodic dynamic system there, whereas all the velocity fields, in particular P, Q, and R, although different to each other, give the same values of average kinetic energy and dissipation rate in V. This is the onset of the fully developed turbulence.

As far as *L* and *U* are concerned, these are both functionals of u following Equation (7), accordingly their variations in terms of $\nu^{-1}$ are peculiar, with quite different results with respect to $u_{1}$ and *ℓ*. In particular, the structure of the first three bifurcations do not show important differences with respect to $u_{1}$ and *ℓ*, whereas, after the third bifurcation ($\nu^{-1}>\nu_{3}^{-1}$), the chaotic regime begins and the bifurcation tree of *U* and *L* exhibits a completely different shape to the corresponding zone of $u_{1}$ and *ℓ*. In detail, the chaotic region extension of *U* and *L* appears to be more limited than that of $u_{1}$ and *ℓ* until to collaps in the lines A–B when $\nu^{-1}>\nu_{A}^{-1}$. This is because the several bifurcations in $\nu_{3}^{-1}<\nu^{-1}<\nu_{A}^{-1}$ correspond to a large number of solutions that show different levels of average kinetic energy and dissipations rate in V which are in some way comparable to each other, respectively. Hence, although the chaotic regime is characterized by myriad of values of $u_{1}$ and *ℓ* which widely sweep the corresponding ranges, *L* and *U*, being related to average kinetic energy and dissipation rate, will exhibit smaller variations. For relatively high values of $\nu^{-1}$, when the velocity fluctuations behavior is ergodic, the averages calculated on phase trajectory tends to the spatial averages. The region of chaotic regime collaps into the line A–B there. Along such lines, for assigned ν, all the solutions—in particular P, Q and R—will exhibit the same level of kinetic energy and dissipation and this represents the regime of fully developed turbulence.

**Figure 2:**
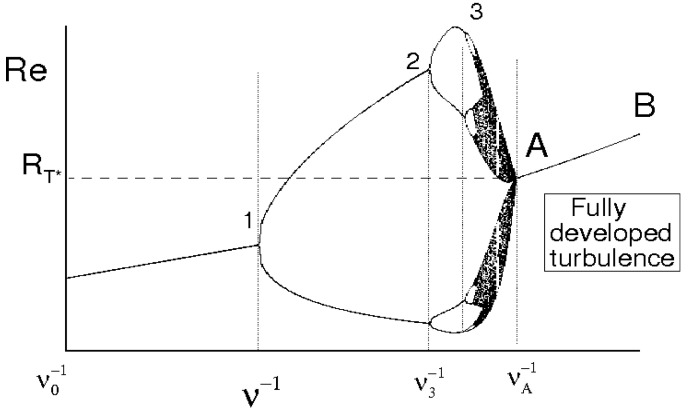
Qualitative scheme of the route toward the turbulence: Reynolds number in terms of kinematic viscosity.

The Reynolds number $Re=\nu^{-1}U\;L$ is shown in terms of $\nu^{-1}$ in Figure 2. Also this map is non universal as it depends on $\nu_{0}^{-1}$. Nevertheless, such representation allows to identify the critical Reynolds number $Re_{*}=R_{T*}=\nu_{*}^{-1}U_{*}\;L_{*}$, the minimum value of $R_{T}$ for which the flow maintains statistically homogeneous and isotropic compatible with $\lambda_{NS}\geq 0$. Hence, a critical Reynolds number $Re_{*}=R_{T*}$ will assume a unique value, represented by the point A of Figure 1 and Figure 2, which plays the role of an universal limit in homogeneous isotropic turbulence. Then, $\nu_{*}\equiv \nu_{A}$, $L_{*}\equiv L_{A}$, $U_{*}=U_{A}$ and the lines A–B represent regimes of fully developed homogeneous isotropic turbulence where

(18) $$\left.\begin{array}{c} L\to \lambda_{T}\\ U\to u\\ Re\to R_{T} \end{array}\right\}\;\;\;\mathrm{along}\;A–B$$

We conclude this section by remarking that the characteristic length of the problem is an undefined quantity in infinite domain. Therefore, the length scales of u are used for determining the flow Reynolds number the critical value of which, $Re_{*}=\nu_{*}^{-1}U_{*}\;L_{*}$ has to be properly estimated. Accordingly, $L_{*}\equiv \lambda_{T*}$ and $U_{*}\equiv u_{*}$, linked with each other, will depend on $R_{T*}$ and ν.

Such qualitative analysis is here used as background to formulate a specific bifurcation analysis of the velocity correlation equation and to determine an estimate of the critical Reynolds number $R_{T*}$.

## 5. Kinematic Bifurcations. Bifurcation Rate

The Navier–Stokes bifurcations have significant implications for what concerns the relative kinematics of velocity field. This kinematics is described by the separation vector ξ (finite scale Lyapunov vector), which satisfies the following equations

(19) $$\begin{array}{c} \begin{array}{c} \dot{x}=u\left(t,x\right),\\ \dot{\xi }=u\left(t,x+\xi \right)-u\left(t,x\right), \end{array} \end{array}$$

being x(t) and y(t)=x(t)+ξ(t) two fluid particles trajectories. In the case of contiguous trajectories, |ξ|→ 0, and Equation (19) read as

(20) $$\begin{array}{c} \begin{array}{c} \dot{x}=u\left(t,x\right),\\ d\dot{x}=\nabla_{x}u\left(t,x\right)dx, \end{array} \end{array}$$

where dx and $\nabla_{x}u\left(t,x\right)$ are, respectively, elemental separation vector and velocity gradient. One point of the physical space is of bifurcation for the velocity field (kinematic bifurcation) if $\nabla_{x}u\left(t,x\right)$ has at least an eigenvalue with zero real part and this happens when its determinant vanishes, that is,

(21) $$\det\left(\nabla_{x}u\left(t,x\right)\right)=0.$$

As seen, when $R_{T}>R_{T*}$, due to Navier–Stokes bifurcations, the velocity field evolution will be characterized by continuous distributions of length scales and velocity values. Therefore, for t>0, the velocity gradient field will exhibit nonsmooth spatial variations where $\langle \nabla_{x}u\left(t,x\right)\rangle =0$, and its determinant, $\det\left(\nabla_{x}u\left(t,x\right)\right)$, is expected to frequently vanish along fluid particles trajectories. To justify this, one could search a link between such property and the statistics of the eigenvalues of $\nabla_{x}u$ which directly arises from the fluid incompressibility [52]. In this regard, observe that an arbitrary particle trajectory $l_{t}:x\left(t\right)$ belongs to the surface $\Sigma_{1}$

(22) $$\Sigma_{1}:\Psi_{1}\left(t;x,y,z\right)\equiv \nabla_{x}\cdot u\left(t,x\right)=0$$

and identically satisfies the equation

(23) $$l_{t}\in \Sigma_{1}:\frac{\partial \Psi_{1}}{\partial t}+\nabla_{x}\Psi_{1}\cdot \dot{x}=0$$

**Figure 3:**
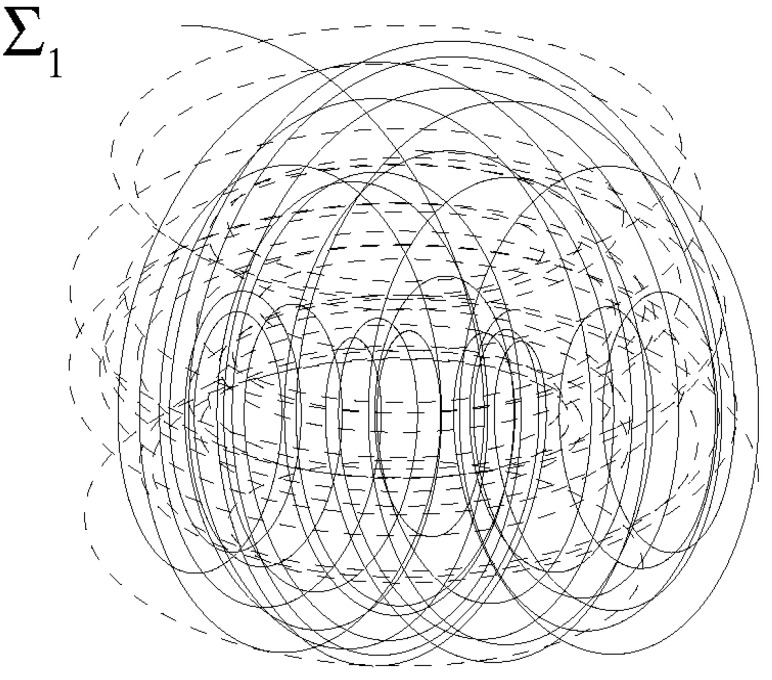
Qualitative scheme of fluid particle trajectory $l_{t}$, bifurcation line $l_{b}$ and their intersections over $\Sigma_{1}$.

Thanks to Navier–Stokes bifurcations and fully developed turbulence hypothesis, for t> 0, $\Sigma_{1}$ and $l_{t}$ will show abrupt variations in their local placement, orientation and curvatures and will tend to sweep the entire physical space. On the other hand, the vanishing condition of velocity gradient determinant

(24) $$\Sigma_{2}:D\left(t;x,y,z\right)\equiv \det\left(\nabla_{x}u\left(t,x\right)\right)=0.$$

defines the surface $\Sigma_{2}\neq \Sigma_{1}$. Thus, the points which satisfy both the conditions (22) and (24) belong to the line $l_{b}=\Sigma_{1}\cap \Sigma_{2}$, and represent all the possible kinematic bifurcations which could happen along $l_{t}$. Because of fully developed turbulence, $l_{b}$ will also show nonsmooth spatial variations and will tend to describe the entire physical space. Therefore, the kinematic bifurcations that occur along $l_{t}$ are obtained as $l_{t}\cap l_{b}$, being $l_{t},l_{b}\in \Sigma_{1}$. As $l_{t}$ and $l_{b}$ are two different curves of the same surface $\Sigma_{1}$ that exhibit chaotic behaviors, their intersections are expected to be very frequent, forming a highly numerous set of points on $\Sigma_{1}$ according to the qualitative scheme of Figure 3 wherein $l_{t}$ and $l_{b}$ are represented by solid and dashed lines. Specifically, for $R_{T}$ > $R_{T*}$, t> 0, the Navier–Stokes bifurcations produce the regime of fully developed turbulence, where length scales and velocity values are continuously doubled and this causes situations where the number of the intersections between $l_{t}$ and $l_{b}$ (kinematic bifurcations) diverges. To show this, the kinematic bifurcation rate is now introduced. This quantity, calculated along a fluid particle trajectory, is defined as follows:

(25) $$\begin{array}{c} \begin{array}{c} S_{b}=\lim_{T\to \infty }\frac{1}{T}\int_{0}^{T}\delta \left(D\right)\;\left|\frac{DD}{Dt}\right|\;dt,\\ \frac{DD}{Dt}=\frac{\partial D}{\partial t}+\nabla_{x}D\cdot u \end{array} \end{array}$$

The rate $S_{b}$ can be much greater than the eigenvalues modulus of $\nabla_{x}u$ and than its maximal Lyapunov exponent. In fact, due to the Navier–Stokes bifurcations and to the hypothesis of fully developed chaos, the characteristic scales of u are continuously doubled, thus $D\equiv \det\left(\nabla_{x}u\right)$ is expected to be a function of the kind

(26) $$\begin{array}{c} \det\left(\nabla_{x}u\right)=D\left(y_{1},y_{2},\ldots ,y_{n}\right),\\ y_{k}=\frac{x}{\ell_{k}},\;k=1,2,\ldots ,n\\ \ell_{1}>\ell_{2}>\ldots >\ell_{n},\\ O|\frac{\partial D}{\partial y_{1}}\left|\approx O\right|\frac{\partial D}{\partial y_{2}}\left|\ldots \approx O\right|\frac{\partial D}{\partial y_{n}}|, \end{array}$$

where, due to bifurcations, *n* tends to diverge and

(27) $$\begin{array}{c} \begin{array}{c} \nabla_{x}D=\sum_{k=1}^{n}\frac{\partial D}{\partial y_{k}}\frac{1}{\ell_{k}},\\ O\left(\frac{1}{\ell_{n}}\;\left|\frac{\partial D}{\partial y_{n}}\cdot u\right|\right)>>>O\left(|\frac{\partial D}{\partial t}|\right) \end{array} \end{array}$$

For one assigned velocity field, from Equations (25) and (26), the simultaneous values of u and $\nabla_{x}\left(\det\left(\nabla_{x}u\right)\right)$ can cause very frequent kinematic bifurcations whose rate can be significantly greater than the maximal Lyapunov exponent of Equation (19) or (20). In fact, following Equations (25) and (26), the order of magnitude of $S_{b}$ identifies the ratio (large scale velocity)–(small scale length)

(28) $$S_{b}\approx \frac{u}{\ell_{n}}$$

where the small scale $\ell_{n}$ represents the minimum distance between to successive kinematic bifurcations encountered along fluid particle trajectory. This means that the changing rate of $\nabla_{x}u$ along $l_{t}$ can be much more rapid than the rate of divergence of two contiguous trajectories.

At this stage of the present study, $S_{b}$ is assumed to be much greater than the maximal Lyapunov exponent of Equation (19) and its estimation will be performed in the following as soon as $\ell_{n}$ is identified by means of this analysis.

## 6. Lyapunov Kinematic Analysis

The aim of this section is to discuss how, in fully developed turbulence, the fluctuations of fluid particles displacements and local strain can be much more rapid and statistically independent with respect to the time variations of velocity field. To analyze this, consider that, in fully developed turbulence, the Navier–Stokes bifurcations cause non smooth spatial variations of u(t,x) which in turn deternine very frequent kinematic bifurcations. Due to the fluid incompressibility, two fluid particles will describe chaotic trajectories, x(t) and y(t)=x(t)+ξ(t), which diverge with each other with a local rate of divergence quantified by the local Lyapunov exponent of finite scale ξ

(29) $$\begin{array}{c} \tilde{\lambda }=\frac{\dot{\xi }\cdot \xi }{\xi \cdot \xi } \end{array}$$

According to such definition of $\tilde{\lambda }$, around to a given instant, $t_{0}$, ξ and $\dot{\xi }$ can be expressed as

(30) $$\begin{array}{c} \begin{array}{c} \xi =Q\left(t\right)\xi \left(t_{0}\right)\exp\left(\tilde{\lambda }\left(t-t_{0}\right)\right),\\ \dot{\xi }=\tilde{\lambda }\xi +\omega_{E}\times \xi \end{array} \end{array}$$

as long as $|\xi |\approx |\xi (t_{0})|=r$, where Q is an orthogonal matrix giving the orientation of ξ with respect to the inertial frame R and $\omega_{E}$ is the angular velocity of ξ with respect to R whose determination is carried out by means of a proper orthogonalization procedure of the Lyapunov vectors described in Reference [6]. The classical local Lyapunov exponent is obtained for |ξ|→ 0, $\tilde{\lambda }\to \Lambda$, that is

(31) $$\tilde{\Lambda }=\frac{dx\cdot \nabla_{x}udx}{dx\cdot dx}$$

On the other hand, dx can be expressed through Equation (20) as follows

(32) $$dx=\exp\left(\int_{0}^{t}\nabla_{x}u\left(t^{\prime },x\left(t^{\prime }\right)\right)dt^{\prime }\right)dx_{0}$$

where the exponential denotes the series expansion of operators

(33) $$\exp\left(\int_{0}^{t}\nabla_{x}u\left(t^{\prime },x\left(t^{\prime }\right)\right)dt^{\prime }\right)=I+\int_{0}^{t}\nabla_{x}u\left(t^{\prime },x\left(t^{\prime }\right)\right)dt^{\prime }+\ldots$$

Although in developed turbulence the Navier–Stokes bifurcations cause abrupt spatial variations of velocity and temperature, with $\lambda_{NS}>$ 0, due to fluid dissipation, u and ϑ are in any case functions of slow growth of t∈(0,∞), whereas ξ and dx, being not bounded by the dissipation effects, are functions of exponential growth of *t*. Therefore, in line with the analysis of Reference [3], and taking into account that $S_{b}>>\sup\left\{\tilde{\lambda }\right\}$, that ξ and dx are much more rapid than u(t,x) being $\sup\left\{\tilde{\lambda }\right\}>>\lambda_{NS}$, it follows that ξ and dx will exhibit power spectra in frequency intervals which are completely separated with respect to those of the power spectum of u. To study this, consider now the Taylor series expansion of u with respect to *t* of the trajectories equations, that is,

(34) $$\begin{array}{c} \dot{x}=u\left(0,x\left(t\right)\right)+\ldots ,\\ \dot{\xi }=u\left(0,x\left(t\right)+\xi \left(t\right)\right)-u\left(0,x\left(t\right)\right)+\ldots ,\;\;\;\mathrm{for}\;\mathrm{finite}\;\mathrm{scale}\;|\xi |,\\ d\dot{x}=\nabla_{x}u\left(0,x\left(t\right)\right)dx+\ldots ,\;\;\;\mathrm{for}\;\mathrm{contiguous}\;\mathrm{trajectories} \end{array}$$

The first terms (terms of 0 order) of such Taylor series do not correspond to time variations in velocity field, thus these do not modify the fluid kinetic energy. Furthermore, as $\sup\left\{\tilde{\lambda }\right\}>>\lambda_{NS}$ (fully developed turbulence), such terms reproduce the particles trajectories as long as $0<t<O(1/\lambda_{NS})$, that is

(35) $$\begin{array}{c} \dot{x}≃u\left(0,x\left(t\right)\right),\\ \dot{\xi }≃u\left(0,x\left(t\right)+\xi \left(t\right)\right)-u\left(0,x\left(t\right)\right),\;\;\;\mathrm{for}\;\mathrm{finite}\;\mathrm{scale}\;|\xi |,\;\;\;\forall t\in \left(0,a\right),\;\;\;a=O\left(\frac{1}{\lambda_{NS}}\right)\\ d\dot{x}≃\nabla_{x}u\left(0,x\left(t\right)\right)dx,\;\;\;\mathrm{for}\;\mathrm{contiguous}\;\mathrm{trajectories} \end{array}$$

Following Equation (35), the fluctuations of ξ and dx are statistically independent with respect to the time variations of the velocity field. Next, $\sup\left\{\tilde{\lambda }\right\}>>\lambda_{NS}$, thus the number of kinematic bifurcation, which happen for $0<t<O(1/\lambda_{NS})$, is expected to be quite high and can be considered to be significative from the statistical point of view.

Now, according to the mathematical analysis of the continuum media [53], the following map is considered

(36) $$\chi \left(.,t\right):x_{0}\to x\left(t\right)$$

which expresses the placement of material elements at the current time *t* in function of their referential position, say $x_{0}=x\left(0\right)$ [53]. From Equation (32), the local fluid strain $\partial x\left(t\right)/\partial x_{0}$ is then an exponential growth function of *t* which, thanks to the above mentioned property of independence of dx from u(t,x), results to be independent and much faster with respect to the time variations of the velocity field. In fact, from the Lyapunov theory of kinematic field, such strain reads as

(37) $$\frac{\partial x}{\partial x_{0}}\equiv \exp\left(\int_{0}^{t}\nabla_{x}u\left(t^{\prime },x\left(t^{\prime }\right)\right)dt^{\prime }\right)\equiv \exp\left(\int_{0}^{t}\nabla_{x}u\left(0,x\left(t^{\prime }\right)\right)dt^{\prime }\right)+\ldots =G\exp\left(\tilde{\Lambda }\;t\right),$$

where G is a proper fluctuating matrix whose elements $G_{ij}=O\left(1\right)$ are functions of of slow growth of *t*. As long as t∈(0,a) we have

(38) $$\frac{\partial x}{\partial x_{0}}≃\exp\left(\int_{0}^{t}\nabla_{x}u\left(0,x\left(t^{\prime }\right)\right)dt^{\prime }\right)=G\exp\left(\tilde{\Lambda }\;t\right),\;\;\;\;\forall t\in \left(0,a\right)$$

that is $\partial x\left(t\right)/\partial x_{0}$ is independent of the time variations of the velocity field.

In brief, as $\sup\left\{\tilde{\lambda }\right\}>>\lambda_{NS}$, two time scales are here considered: one associated with the velocity field and the other one related to the relative fluid kinematics. Thus, ξ, $\partial x\left(t\right)/\partial x_{0}$ and $\tilde{\lambda }$ are statistically independent of u. Furthermore, due to very frequent kinematic bifurcations in $\left(t,t+1/\lambda_{NS}\right)$, ξ, local strain and $\tilde{\lambda }$ are expected to be continuously distributed in their variation ranges. This conclusion is supported by the arguments in References [54,55] (and references therein), where the author remarks among other things that the fields u(t,x), (and therefore also u(t,x+ξ)−u(t,x)) produce chaotic trajectories also for relatively simple mathematical structure of u(t,x) (also for steady fields!).

## 7. *Turbulent Energy Cascade, Material Vorticity and Link with Classical Kinematic Lyapunov Analysis

By means of theoretical considerations based on the classical Lyapunov theory and on the property that the kinematic bifurcation rate is much larger than the maximal Lyapunov exponent of the velocity gradient, an interpretation of the kinetic energy cascade phenomenon is given which shows that η≡dx is much more rapid and statistically independent with respect to u. Following such considerations, the vorticity equation of a material element (material vorticity)—directly obtained making the curl of the incompressible Navier–Stokes equations—is compared with the evolution equation of η which follows the classical Lyapunov theory. These equations read as

(39) $$\begin{array}{c} \frac{D\omega }{Dt}\equiv \frac{\partial \omega }{\partial t}+\nabla_{x}\omega \;u=\nabla_{x}u\;\omega +\nu \nabla_{x}^{2}\omega ,\\ \mathrm{being}\;\omega =\nabla_{x}\times u,\\ \frac{D\eta }{Dt}\equiv \dot{\eta }=\nabla_{x}u\;\eta ,\\ \frac{Dx}{Dt}\equiv \dot{x}=u\left(t,x\right),\\ t\in \left(t_{0},t_{0}+a\right) \end{array}$$

From such relations, it is apparent that, for inviscid fluids (ν=0), the time variations of η and of ω along a fluid particle trajectory *x*=x(t) follow the same equation, thus ω identifies those particular Lyapunov vectors such that $\eta \propto \nabla_{x}\times u$ at the initial time $t_{0}$. On the other hand, regardless of the initial condition η(0), η(t) tends to align with the direction of the maximum rising rate of the trajectories distance [56]. If $\omega \left(t_{0}\right)=k\;\eta \left(t_{0}\right)$, then ω(t)=kη(t), $\forall t>t_{0}$ (von Helmholtz), where *k* does not depend on *t*, while η is a fast growth function of *t*. Thus, following the Lyapunov theory, for inviscid fluids, |ω|, calculated along *x* = x(t), tends to exponentially rise with *t*. More in general, for inviscid fluids, ω and η are both fast growth (exponential) functions of the time, where ω tends to align to the direction of maximum growth rate of |η| [56].

A nonzero viscosity influences the time variations of the material vorticity making this latter a slow growth function of $t\in (t_{0},\infty )$, whereas η and ξ remain in any case exponential growth functions of *t*. This implies that, for ν≠0, the characteristic time scales of u (and ϑ) and η are different and that after the time $t_{0}+a$, the fluctuations of ξ result in being statistically independent from u. This holds also when ν→0 for properly small length scales, except for ν=0.

Based on the previous observations, the combined effect of very frequent bifurcations and stretching term $\nabla_{x}u\;\omega$ produces the kinetic energy cascade. This phenomenon regards each fluid particle, where $\nabla_{x}u\;\omega$ acts on the material vorticity in the same way in which $\nabla_{x}u\;\eta$ influences η. In fact, according to Equation (39), as long as $|\nabla_{x}u\;\omega |>>\nu |\nabla_{x}^{2}\omega |$, arbitrary material lines η—thus arbitrary material volumes built on different Lyapunov vectors η, that is, $\eta_{1}\times \eta_{2}\cdot \eta_{3}$—moving along x(t), experience the material vorticity growth and deform according to the Lyapunov theory. According to the analysis of the previous section, such growth phenomenon, due to $\nabla_{x}u\;\omega$, preserves the average kinetic energy and corresponds to the continuous kinetic energy transfer from large to small scales that is, the kinetic energy cascade phenomenon. Due to the arbitrary choice of x(t), this pertains to all the fluid particles. For what concerns the thermal energy cascade, ϑ is a passive scalar, the temperature follows the velocity fluctuations according to Equation (15), thus the cascade of thermal energy is direct consequence of the mechanism of kinetic energy cascade.

In brief, the energy cascade can be linked to the material vorticity tendency to be proportional to the classical Lyapunov vectors whose modulus changes according to the Lyapunov theory. Specifically, according to Equation (39) and taking into account that $S_{b}>>\sup\left\{\tilde{\lambda }\right\}$, η is much faster and statistically independent with respect to the velocity field, while the energy cascade can be viewed as a continuous and intensive stretching and folding process of fluid particles which involves smaller and smaller length scales during their motion and where the particle folding process happens with a frequency given by the bifurcation rate.

## 8. Distribution Functions of u, ϑ, *x*, ξ and $\tilde{\lambda }$

Following the present formulation, u, ϑ, *x* and ξ are the fluid state variables. Therefore, the distribution function of u, ϑ, *x* and ξ, say *P*, varies according to the Liouville theorem associated with (4), (5) and (19) [57]

(40) $$\frac{\partial P}{\partial t}+\frac{\delta }{\delta u}\cdot \left(P\dot{u}\right)+\frac{\delta }{\delta \vartheta }\cdot \left(P\dot{\vartheta }\right)+\frac{\partial }{\partial x}\cdot \left(P\dot{x}\right)+\frac{\partial }{\partial \xi }\cdot \left(P\dot{\xi }\right)=0$$

where, according to the notation of Equations (4) and (5), δ/δu and δ/δϑ are functional partial derivatives with respect to u and ϑ, respectively and $\left(\partial /\partial \circ \right)\cdot$ stands for the divergence with respect to ∘. In line with the previous analysis and with References [5,6], *P* can be factorized as follows

(41) $$P\left(t,u,\vartheta ,x,\xi \right)=F\left(t,u,\vartheta \right)P_{\xi }\left(t,x,\xi \right)$$

being *F* and $P_{\xi }$ the distribution functions of (u, ϑ) and of (*x*, ξ), respectively. It is worth remarking that Equation (41) represents the crucial point of this analysis, being the hypothesis of fully developed turbulence following the present formulation. The evolution equations of *F* and $P_{\xi }$ are formally obtained from Equation (40) and taking into account the aforementioned statistical independence (41). This allows to split the Liouville Equation (40) in the two following equations

(42) $$\begin{array}{c} \begin{array}{c} \frac{\partial F}{\partial t}+\frac{\delta }{\delta u}\cdot \left(F\dot{u}\right)+\frac{\delta }{\delta \vartheta }\cdot \left(F\dot{\vartheta }\right)=0,\\ \frac{\partial P_{\xi }}{\partial t}+\frac{\partial }{\partial x}\cdot \left(P_{\xi }\dot{x}\right)+\frac{\partial }{\partial \xi }\cdot \left(P_{\xi }\dot{\xi }\right)=0 \end{array} \end{array}$$

where the boundary conditions of $P_{\xi }$ read as

(43) $$P_{\xi }=0,\;\forall \left(x,\xi \right)\in \partial \left\{\left\{x\right\}\times \left\{\xi \right\}\right\}$$

In case of homogeneous and isotropic turbulence, $P_{\xi }$ does not depend on *x* and can be expressed in function of the finite scale *r* as follows

(44) $$\begin{array}{c} \begin{array}{c} P_{\xi }\approx \sum_{k}\delta \left(\xi -r_{k}\right),\\ |r_{k}|=r,\forall k \end{array} \end{array}$$

where δ denotes the Dirac’s delta and $r_{k}$ are uniformly distributed points on a sphere S of radius *r* due to isotropy hypothesis, being *k* a generic index indicating the several points on S. This leads to

(45) $$P_{\xi }=\frac{1}{4\pi r^{2}}\;\delta \left(|\xi |-r\right)=\left\{\begin{array}{c} C\to \infty \;\mathrm{if}\;|\xi |=r\\ 0\;\mathrm{elsewhere} \end{array}\right.$$

Also $\tilde{\lambda }$ and $\omega_{E}$ are statistically independent of the velocity field and are continuously distributed in their ranges of variation. In particular, the PDF of $\tilde{\lambda }$, say $P_{\lambda }$, can be calculated by means of $P_{\xi }$ with the Frobenius–Perron equation

(46) $$P_{\lambda }\left(\tilde{\lambda }\right)=\int_{x}\int_{\xi }P_{\xi }\;\delta \left(\tilde{\lambda }-\frac{\dot{\xi }\cdot \xi }{\xi \cdot \xi }\right)\;dxd\xi$$

Now, in isotropic turbulence, the longitudinal component of the velocity difference $\dot{\xi }\cdot \xi /r$ is uniformely distributed in its variation range as ξ sweeps S, while, due to the fluid incompressibility, $\tilde{\lambda }$ is expected to vary in the interval $\left(-\lambda_{S}/2,\lambda_{S}\right)$, where $\lambda_{S}=\sup\left\{\tilde{\lambda }\right\}$. Therefore, substituting Equation (44) in Equation (46), we found that $\tilde{\lambda }$ uniformely sweeps $\left(-\lambda_{S}/2,\lambda_{S}\right)$, according to

(47) $$P_{\lambda }=\left\{\begin{array}{c} \frac{2}{3}\frac{1}{\lambda_{S}},\;\mathrm{if}\;\tilde{\lambda }\in \left(-\frac{\lambda_{S}}{2},\lambda_{S}\right)\\ 0\;\mathrm{elsewhere} \end{array}\right.$$

Observe that Equations (45) and (47) agree with the results of Reference [6], where the author shows that ξ and $\tilde{\lambda }$ are both uniformely distributed in their ranges by means of the condition H = max compatible with certain constraints, being H the entropy associated with the kinematic state (x,ξ). This is because the isotropic homogeneous turbulence hypotheses, here expressed through Equations (44) and (45), correspond to the maximum of H. The causes of the nonsymmetric distribution of $\tilde{\lambda }$ with respect to the origin, also analyzed in Reference [6], are fluid incompressibility and alignment property of ξ with respect to the maximum rising rate direction. Following such property, regardless of the initial condition ξ(0), ξ(t) tends to align with the direction of the maximum rising rate of the trajectories distance [56]. Therefore, such a distribution function provides positive average Lyapunov exponents and gives the link between average and square mean values of the finite scale Lyapunov exponent according to

(48) $${\langle \tilde{\lambda }\rangle }_{\xi }=\frac{1}{2}\sqrt{{\langle {\tilde{\lambda }}^{2}\rangle }_{\xi }}>0.$$

where ${\langle \circ \rangle }_{\xi }$ indicates the average of ∘ calculated, through $P_{\xi }$ or $P_{\lambda }$.

## 9. *Finite Time Lyapunov Exponents and Their Distribution in Fully Developed Turbulence

Altough the local Lyapunov exponent $\tilde{\lambda }$ quantifies the local trajectories divergence in a point of space, in practice, the trajectory stability is evaluated by observing the particle motion in a finite time interval, say $\left(t_{0},t_{0}+\tau \right)$. For this reason, it is useful to define the finite time Lyapunov exponent as the average of $\tilde{\lambda }$ in such time interval, that is

(49) $$\begin{array}{c} {\tilde{\lambda }}_{\tau }=\frac{1}{\tau }\int_{t_{0}}^{t_{0}+\tau }\tilde{\lambda }\;dt=\frac{1}{\tau }\int_{t_{0}}^{t_{0}+\tau }\frac{d}{dt}\ln\varrho \;dt=\frac{1}{\tau }\ln\left(\frac{\varrho (t_{0}+\tau )}{\varrho (t_{0})}\right).\\ \varrho =|\xi |. \end{array}$$

This exponent trivially satisfies

(50) $$\lim_{\tau \to 0}{\tilde{\lambda }}_{\tau }=\tilde{\lambda }.$$

If τ is properly high, a statistically significant number of kinematic bifurcations *n* can occur for $t\in (t_{0},t_{0}+\tau )$, thus ${\tilde{\lambda }}_{\tau }$ is in general a fluctuating variable which exhibits variations whose amplitude diminishes as τ increases. Accordingly, ${\tilde{\lambda }}_{\tau }$ will be distributed following a Gaussian PDF in fully developed turbulence. In fact, due to the bifurcations encountered in $\left(t_{0},t_{0}+\tau \right)$, ${\tilde{\lambda }}_{\tau }$ can be written as sum of several terms, each of them related to the effects of a single bifurcation, that is,

(51) $${\tilde{\lambda }}_{\tau }=\frac{1}{\tau }\ln\left(\frac{\varrho (t_{0}+\tau )}{\varrho (t_{0})}\right)=\frac{1}{\tau }\ln\left(\frac{\varrho (t_{0}+\tau )}{\varrho_{n-1}}\;\frac{\varrho_{n-1}}{\varrho_{n-2}}\;\ldots \;\frac{\varrho_{1}}{\varrho (t_{0})}\right)=\frac{1}{\tau }\sum_{k=1}^{n}\ln\left(\frac{\varrho_{k}}{\varrho_{k-1}}\right)$$

where $\ln(\varrho_{k}/\varrho_{k-1})$ gives the contribution of the *k*th bifurcation starting from $t_{0}$, being $\varrho_{k-1}$ and $\varrho_{k}$ the Lyapunov vectors moduli calculated immediately before and after the *k*th bifurcation. On the other hand, due to fully developed chaos, each of such terms is expected to be statistically independent of all other ones and if τ→∞, the number of encountered bifurcations *n* diverges. Hence, a proper variant of the central limit theorem can be applied and this would guarantee that ${\tilde{\lambda }}_{\tau }$ tends to a Gaussian stochastic variable [58]. The novelty of the present section consists in the implication that the property $S_{b}>>\lambda_{\tau }$ has on Equation (51). Such property should ensure that $\lambda_{\tau }$ can be approximated to a gaussian stochastic variable also for certain finite values of τ. In fact, if $\tau \approx 1/\lambda_{\tau }$ or $\tau ≳1/\lambda_{\tau }$, the time interval $\left(t_{0},t_{0}+\tau \right)$ should include a statistically significant number of kinematic bifurcations, thus the distribution function of $\lambda_{\tau }$ is expected to be a Gaussian PDF, expecially for relatively high values of the Taylor scale Reynolds number.

## 10. Closure of von Kármán–Howarth and Corrsin Equations

Starting from the property of statistical independence (41) and adopting the Liouville theorem, the closure formulas of von Kármán-Howarth and Corrsin equations are here determined and the effects of the chaotic trajectories divergence on these closures are discussed.

In fully developed isotropic homogeneous turbulence, the pair correlation functions of longitudinal velocity components and of temperature, defined as

(52) $$\begin{array}{c} f\left(r\right)=\frac{\langle u_{r}\left(t,x\right)u_{r}\left(t,x+r\right)\rangle }{u^{2}}\equiv \frac{\langle u_{r}u_{r}^{\prime }\rangle }{u^{2}},\\ f_{\theta }\left(r\right)=\frac{\langle \vartheta (t,x)\vartheta (t,x+r)\rangle }{\theta^{2}}\equiv \frac{\langle \vartheta \vartheta^{\prime }\rangle }{\theta^{2}}. \end{array}$$

satisfy the von Kármán–Howarth equation [17] and Corrsin equation [19,20], respectively, where

(53) $$u_{r}=u\left(t,x\right)\cdot \frac{r}{r},\;u_{r}^{\prime }=u\left(t,x+r\right)\cdot \frac{r}{r}$$

von Kármán–Howarth and Corrsin equations are properly obtained from the Navier–Stokes and heat equations written in two points of space, say x and x+r. These correlation equations read as follows

(54) $$\begin{array}{c} \frac{\partial f}{\partial t}=\frac{K}{u^{2}}+2\nu \left(\frac{\partial^{2}f}{\partial r^{2}}+\frac{4}{r}\frac{\partial f}{\partial r}\right)+\frac{10\nu }{\lambda_{T}^{2}}f,\\ \frac{\partial f_{\theta }}{\partial t}=\frac{G}{\theta^{2}}+2\chi \left(\frac{\partial^{2}f_{\theta }}{\partial r^{2}}+\frac{2}{r}\frac{\partial f_{\theta }}{\partial r}\right)+\frac{12\chi }{\lambda_{\theta }^{2}}f_{\theta }, \end{array}$$

The boundary conditions associated with such equations are

(55) $$\begin{array}{c} f\left(0\right)=1,\;\;\;\lim_{r\to \infty }f\left(r\right)=0,\\ f_{\theta }\left(0\right)=1,\;\;\;\lim_{r\to \infty }f_{\theta }\left(r\right)=0, \end{array}$$

being $u\equiv \sqrt{\langle u_{r}^{2}\rangle }$, $\theta \equiv \sqrt{\langle \vartheta^{2}\rangle }$, where $\lambda_{T}\equiv \sqrt{-1/f^{\prime \prime }\left(0\right)}$ and $\lambda_{\theta }\equiv \sqrt{-2/f_{\theta }^{\prime \prime }\left(0\right)}$ are Taylor and Corrsin microscales, respectively. The quantities *K* and *G*, arising from inertia forces and convective terms, give the energy cascade and are expressed as [17,19,20]

(56) $$\begin{array}{c} \left(3+r\frac{\partial }{\partial r}\right)K=\frac{\partial }{\partial r_{k}}\langle u_{i}u_{i}^{\prime }\left(u_{k}-u_{k}^{\prime }\right)\rangle ,\\ G=\frac{\partial }{\partial r_{k}}\langle \vartheta \vartheta^{\prime }\left(u_{k}-u_{k}^{\prime }\right)\rangle , \end{array}$$

where the repeated index denotes the summation convention. Following the theory [17,19,20], *K* and *G* are linked to the longitudinal triple velocity correlation function *k* and to the triple correlation between $u_{r}$ and ϑ, according to

(57) $$\begin{array}{c} K\left(r\right)=u^{3}\left(\frac{\partial }{\partial r}+\frac{4}{r}\right)k\left(r\right),\;\mathrm{where}\;k\left(r\right)=\frac{\langle u_{r}^{2}u_{r}^{\prime }\rangle }{u^{3}},\\ G\left(r\right)=2u\theta^{2}\left(\frac{\partial }{\partial r}+\frac{2}{r}\right)m^{*}\left(r\right),\;\mathrm{where}\;m^{*}\left(r\right)=\frac{\langle u_{r}\vartheta \vartheta^{\prime }\rangle }{\theta^{2}u}, \end{array}$$

As well known from the literature [17,19,20], without particular hypotheses about the statistics of u and ϑ, *K* and *G* are unknown quantities which can not be expressed in terms of *f* and $f_{\theta }$, thus at this stage of this analysis, both the correlations Equation (54) are not closed.

In order to obtain analytical forms of *K* and *G*, observe that these latter, representing the energy flow between length scales in the fluid, do not modify the total amount of kinetic and thermal energies [18,19]. Indeed, convective term, inertia and pressure forces determine interactions between Fourier components of velocity and temperature fields providing the transfer of kinetic and thermal energy between volume elements in the wavenumber space, whereas the global effect of such these interactions leaves $u^{2}$ and $\theta^{2}$ unaltered [18,19]. On the other hand, the proposed statistical independence property (41) allows to write the time derivative of *P* as sum of two terms

(58) $$\frac{\partial P}{\partial t}=P_{\xi }\frac{\partial F}{\partial t}+F\frac{\partial P_{\xi }}{\partial t}$$

the first one of which, being related to ∂F/∂t, provides the time variations of velocity and temperature fields. The second one, linked to $\partial P_{\xi }/\partial t$, not producing a change of $u^{2}$ and $\theta^{2}$, identifies the energy cascade effect. Therefore, *K* and *G* arise from the second term of (58) and can be expressed, by means of the Liouville theorem (40) and Equation (42), in terms of material displacements ξ, taking into account flow homogeneity and fluid incompressibility. Specifically, from Equations (40)–(42), *K* and *G*, directly arising from $-F\partial (P_{\xi }\dot{\xi })/\partial \xi$, are calculated as follows

(59) $$\begin{array}{c} K=-\int_{U}\int_{\Xi }F\frac{\partial }{\partial \xi }\cdot \left(P_{\xi }\dot{\xi }\right)u_{\xi }u_{\xi }^{*}\;dUd\Xi ,\\ G=-\int_{U}\int_{\Xi }F\frac{\partial }{\partial \xi }\cdot \left(P_{\xi }\dot{\xi }\right)\;\vartheta \vartheta^{*}dUd\Xi , \end{array}$$

where $U=\left\{u\right\}\times \left\{\vartheta \right\}$, $\Xi =\left\{\xi \right\}$ and dU and dΞ are the corresponding elemental volumes, and

(60) $$\begin{array}{c} u_{\xi }=u\left(t,x\right)\cdot \frac{\xi }{\xi },\;\;u_{\xi }^{*}=u\left(t,x+\xi \right)\cdot \frac{\xi }{\xi },\\ \vartheta =\vartheta \left(t,x\right),\;\;\vartheta^{*}=\vartheta \left(t,x+\xi \right), \end{array}$$

Integrating Equation (59) with respect to U, we obtain

(61) $$\begin{array}{c} K=-u^{2}\int_{\Xi }\frac{\partial }{\partial \xi }\cdot \left(P_{\xi }\dot{\xi }\right)f\left(\xi \right)\;d\Xi ,\\ G=-\theta^{2}\int_{\Xi }\frac{\partial }{\partial \xi }\cdot \left(P_{\xi }\dot{\xi }\right)f_{\theta }\left(\xi \right)\;d\Xi , \end{array}$$

Again, integrating by parts Equation (61) with respect to Ξ, taking into account the boundary conditions (43) ($P_{\xi }\equiv$ 0, ∀ξ∈∂Ξ) and the isotropy hypothesis, *K* and *G* are written as

(62) $$\begin{array}{c} \begin{array}{c} K=u^{2}\int_{\Xi }P_{\xi }\frac{\partial f}{\partial \xi }\cdot \dot{\xi }\;d\Xi =u^{2}\int_{\Xi }P_{\xi }\frac{\partial f}{\partial \xi }\frac{\xi }{\xi }\cdot \dot{\xi }\;d\Xi ,\\ G=\theta^{2}\int_{\Xi }P_{\xi }\frac{\partial f_{\theta }}{\partial \xi }\cdot \dot{\xi }\;d\Xi =\theta^{2}\int_{\Xi }P_{\xi }\frac{\partial f_{\theta }}{\partial \xi }\frac{\xi }{\xi }\cdot \dot{\xi }\;d\Xi , \end{array} \end{array}$$

Now, the Lyapunov theory provides $\dot{\xi }$ = $\tilde{\lambda }\xi +\omega_{E}\times \xi$, and in isotropic homogeneous turbulence $P_{\xi }=\delta \left(|\xi |-r\right)/4\pi r^{2}$, thus *K* and *G* are

(63) $$\begin{array}{c} \begin{array}{c} K=u^{2}\int_{\Xi }P_{\xi }\frac{\partial f}{\partial \xi }\xi \tilde{\lambda }\;d\Xi =u^{2}\frac{\partial f}{\partial r}r{\langle \tilde{\lambda }\rangle }_{\xi },\\ G=\theta^{2}\int_{\Xi }P_{\xi }\frac{\partial f_{\theta }}{\partial \xi }\xi \tilde{\lambda }\;d\Xi =\theta^{2}\frac{\partial f_{\theta }}{\partial r}r{\langle \tilde{\lambda }\rangle }_{\xi }, \end{array} \end{array}$$

Furthermore, the finite scale Lyapunov theory also gives the relationship between velocity correlation and Lyapunov exponents according to

(64) $${\langle {\left(u_{\xi }^{*}-u_{\xi }\right)}^{2}\rangle }_{\xi }=2u^{2}\left(1-f(r)\right)={\langle {\tilde{\lambda }}^{2}\rangle }_{\xi }r^{2},$$

where ${\langle \tilde{\lambda }\rangle }_{\xi }$ and ${\langle {\tilde{\lambda }}^{2}\rangle }_{\xi }$ are linked with each other through Equation (48), therefore the closure formulas of *K* and *G* are in terms of autocorrelations and of their gradients

(65) $$\begin{array}{c} \begin{array}{c} K\left(r\right)=u^{3}\sqrt{\frac{1-f}{2}}\frac{\partial f}{\partial r},\\ G\left(r\right)=u\theta^{2}\sqrt{\frac{1-f}{2}}\frac{\partial f_{\theta }}{\partial r}, \end{array} \end{array}$$

These closure formulas do not include second order derivatives of autocorrelations, thus Equation (65) do not correspond to a diffusive model. The energy cascade expressed by Equation (65) is not based on the eddy viscosity concept, being the result of the trajectories divergence in the continuum fluid. This cascade phenomenon and Equation (65) are here interpreted as follows:

(1) In fully developed chaos, the Navier–Stokes bifurcations determine a continuous distribution of velocity, temperature and of length scales, where one single bifurcation causes doubling of velocity, temperature, length scale and of all the properties associated with the velocity and temperature fields according to Equations (15) and (16). This leads to nonsmooth spatial variations of velocity field and very frequent kinematic bifurcations.
(2) The huge kinematic bifurcations rate generates in turn continuous distributions of $\tilde{\lambda }$ and ξ, while fluid incompressibility and the mentioned alignment property of ξ make $\tilde{\lambda }$ unsymmetrically distributed with $\bar{\lambda }\left(r\right)$≡${\langle \tilde{\lambda }\rangle }_{\xi }>$ 0 and the relative particles trajectories to be chaotic.
(3) The tendency of the material vorticity to follow direction and variations of the Lyapunov vectors gives the phenomenon of the kinetic energy cascade.

The main asset of Equation (65) with respect to the other models is that Equation (65) are not based on phenomenological assumptions, such as, for instance, the eddy viscosity paradigm [22,23,24,28,29,33] but are obtained through theoretical considerations concerning the statistical independence of ξ from u and the Liouville theorem.

**Remark** **1.** At this stage of the present analysis, it is worth remarking on the importance of the hypothesis of the statistical independence of u and ξ expressed by Equation (41). This latter, expressing the hypothesis of fully developed turbulence following this study, leads to the analytical expressions of K and G separating the effects of the trajectories divergence in the physical space from those of the velocity field fluctuations in the Navier–Stokes phase space. Without such hypothesis, the energy cascade effect can not be expressed through the term $-F\partial (P_{\xi }\dot{\xi })/\partial \xi$ and using Equation (59), thus the proposed closures (65) cannot be determined.

Thanks to their theoretical foundation, Equation (65) do not exhibit free model parameters or empirical constants which have to be identified. These closure formulas coincide with those just obtained by the author in the previous works [1,4,5]. While References [1,4] derive such closures expressing the local fluid act of motion in the finite scale Lyapunov basis and using the frame invariance property of *K* and *G*, Reference [5] achieves the same formulas adopting maximum and average finite scale Lyapunov exponents, properly defined and the statistical independence of ξ and u. Here, unlike References [1,4,5], Equation (65) are determined exploiting the unsymmetric distribution function of $\tilde{\lambda }$ just studied in Reference [6], showing that the assumptions of References [1,4,5] are congruent with the present analysis, corroborating the results of the previous work.

References [1,4] show that these closures adequately describe the energy cascade phenomenon and the energy spectra. In detail, *K* reproduces the kinetic energy cascade mechanism following the Kolmogorov law and *G* gives the thermal energy cascade in line with the theoretical argumentation of Kolmogorov, Obukhov–Corrsin and Batchelor [35,36,37], with experimental results [38,39] and with numerical data [40,41]. Moreover, Equation (65) allows the calculation of the skewness of $\Delta u_{r}$ and $\partial u_{r}/\partial r$ which is directly linked to the energy cascade intensity. This is [18]

(66) $$H_{3}\left(r\right)\equiv \frac{\langle {\left(\Delta u_{r}\right)}^{3}\rangle }{{\langle {\left(\Delta u_{r}\right)}^{2}\rangle }^{3/2}}=\frac{6k(r)}{{\left(2\left(1-f\left(r\right)\right)\right)}^{3/2}}$$

Then, substituting Equation (65) in Equation (66), the skewness of $\partial u_{r}/\partial r$ is

(67) $$H_{3}\left(0\right)=-\frac{3}{7}$$

This constant quantifies the effect of chaotic relative trajectories on the energy cascade in isotropic turbulence and agrees with the several results obtained through direct numerical simulation of the Navier–Stokes equations (DNS) [59,60,61] (−0.47÷−0.40) and by means of Large–eddy simulations (LES) [62,63,64] (−0.42÷−0.40). For the sake of reader convenience, Table 1 recalls the comparison, presented in Reference [5,6], between the value of the skewness $H_{3}\left(0\right)$ of this analysis and those achieved by the aforementioned works. The results were that the maximum absolute difference between the proposed value and the other results were less than 10%. Therefore, the proposed hypotheses, leading to the distribution function (47) and to the closures (65), seem to be adequate assumptions for estimating turbulent energy cascade and spectra.

We conclude this section by observing the limits of the proposed closures (65). These limits directly derive from the hypotheses under which Equation (65) are obtained: Equation (65) are valid only in a regime of fully developed chaos where the turbulence exhibit homogeneity and isotropy. Otherwise, during the transition through intermediate stages of turbulence or in more complex situations with particular boundary conditions, for instance in the presence of wall, Equation (65) cannot be applied.

## 11. Properties of the Proposed Closures

Here, some of the properties of the proposed closures (65) are renewed, with particular reference to the evolution times of the developed velocity and temperature autocorrelations. In detail, we will show that these correlations reach their developed shape in finite times which depend on the initial condition and that, after this period, the hypothesis of statistical independence could be not more verified. This result is given in Reference [5], where the author adopts a specific Lyapunov analysis using two exponents properly defined. Unlike in Reference [5], such a result is here achieved through the previously obtained local finite scale Lyapunov exponent distribution (47). To analyze this, the evolution equations of *u*, θ, $\lambda_{T}$ and $\lambda_{\theta }$ are first obtained taking the coefficients of order $r^{0}$ and $r^{2}$ of Equation (54) arising from the Taylor series expansion of even powers of *f* and $f_{\theta }$ [17,19,20]

(68) $$\begin{array}{c} \begin{array}{c} f=1-\frac{1}{2}{\left(\frac{r}{\lambda_{T}}\right)}^{2}+\ldots ,\\ f_{\theta }=1-{\left(\frac{r}{\lambda_{\theta }}\right)}^{2}+\ldots , \end{array} \end{array}$$

This leads to the following equations

(69) $$\begin{array}{c} \begin{array}{c} \frac{du^{2}}{dt}=-\frac{10\nu }{\lambda_{T}^{2}}u^{2},\\ \frac{d\theta^{2}}{dt}=-\frac{12\chi }{\lambda_{\theta }^{2}}\theta^{2}, \end{array} \end{array}$$

(70) $$\begin{array}{c} \begin{array}{c} \frac{d\lambda_{T}}{dt}=-\frac{u}{2}+\frac{\nu }{\lambda_{T}}\left(\frac{7}{3}f^{IV}\left(0\right)\lambda_{T}^{4}-5\right),\\ \frac{d\lambda_{\theta }}{dt}=-\frac{u}{2}\frac{\lambda_{\theta }}{\lambda_{T}}+\frac{\chi }{\lambda_{\theta }}\left(\frac{5}{6}f_{\theta }^{IV}\left(0\right)\lambda_{\theta }^{4}-6\right) \end{array} \end{array}$$

While Equation (69) do not depend on the particular adopted closures [17,19,20], Equation (70) are obtained using the proposed closures (65). On the other hand, it is useful to consider the fluctuations of the classical Lyapunov exponent, defined as

(71) $$\begin{array}{c} \begin{array}{c} \tilde{\Lambda }=\lim_{r\to 0}\tilde{\lambda }=\lim_{r\to 0}\frac{d}{dt}\ln\varrho ,\\ \varrho =|\xi | \end{array} \end{array}$$

which are related to *f* through Equations (64) and (68) in such a way that

(72) $$\Lambda =\sqrt{\langle {\tilde{\Lambda }}^{2}\rangle }=\frac{u}{\lambda_{T}}\propto \lim_{r\to 0}\frac{d}{dt}{\langle \ln\varrho \rangle }_{\xi }\approx \left|\frac{d\ln\lambda_{T}}{dt}\right|.$$

being Λ the root mean square of $\tilde{\Lambda }$.

Following Equation (70), the time variations of $\lambda_{T}$, $\lambda_{\theta }$ and Λ are now discussed. The first terms at the R.H.S. of Equation (70) provide the turbulent energy cascade, whereas the other ones arise from the fluid diffusivities. While these latter contribute to increasing both the correlation lengths, the energy cascade mechanism tends to reduce these scales and if such a mechanism is sufficiently stronger than diffusivities, then $d\lambda_{T}/dt<$ 0 and $d\lambda_{\theta }/dt<$ 0.

**Figure 4:**
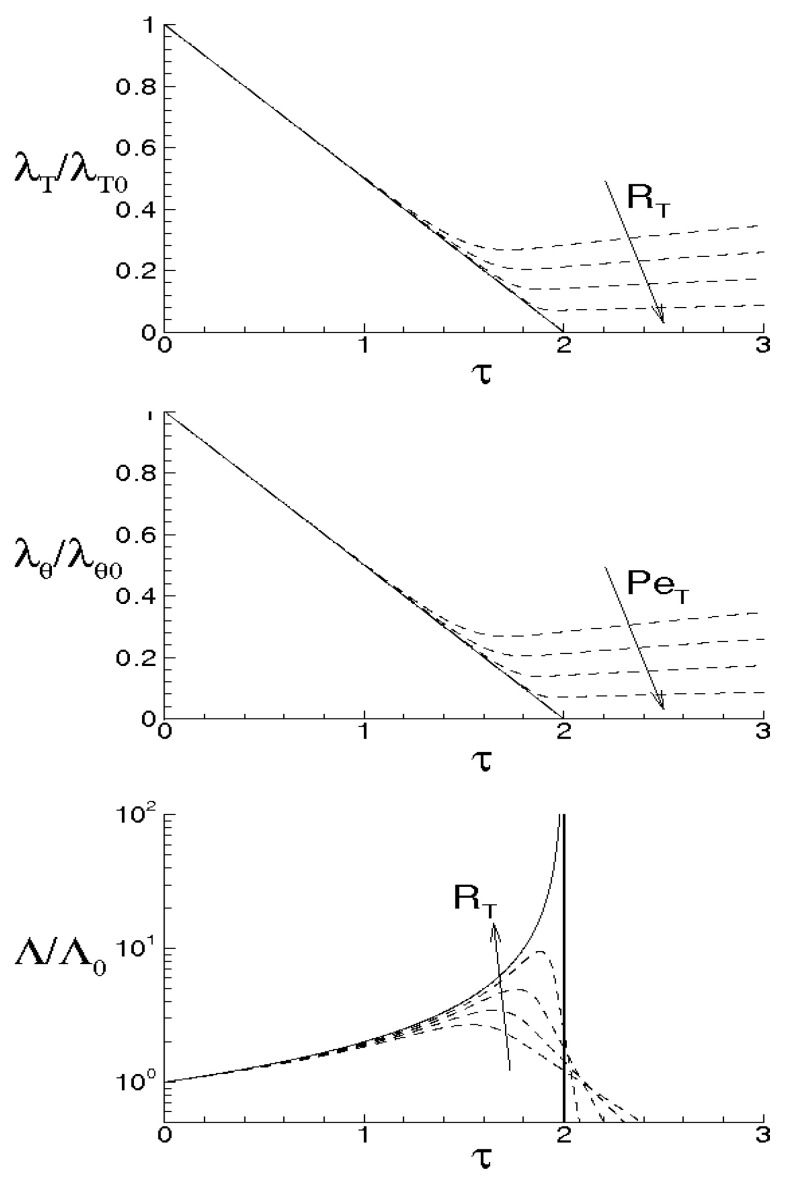
Taylor and Corrsin microscales and root mean square of classical Lyapunov exponent in function of the dimensionless time.

For sake of our convenience, the condition ν=0, χ=0 is first studied. In this case, *u* and θ are both constants, whereas $\lambda_{T}$, $\lambda_{\theta }$ and Λ vary with t. In detail, $\lambda_{T}$ and $\lambda_{\theta }$ are proportional to each other and vary linearly with time according to

(73) $$\begin{array}{c} \begin{array}{c} \frac{\lambda_{T}\left(t\right)}{\lambda_{T}\left(0\right)}\equiv \frac{\lambda_{\theta }\left(t\right)}{\lambda_{\theta }\left(0\right)}=1-\frac{\tau }{2},\\ \frac{\Lambda (t)}{\Lambda (0)}=\frac{1}{1-\tau /2},\\ \tau =t\;\Lambda (0), \end{array} \end{array}$$

while Λ monotonically rises and goes to infinity in a finite time, being τ the dimensionless time. When ν=χ=0, the energy cascade provides that both the microscales decrease until to τ→2, where both the correlations are considered to be fully developed, $\lambda_{T}\to$ 0, $\lambda_{\theta }\to$ 0 and Λ→∞ (see solid lines of Figure 4).

Thus, the two correlations will exhibit developed shapes in finite times whose values depend on the initial condition Λ(0). The meaning that both the microscales are decreasing functions of τ is that kinetic and thermal energies are continuously transferred from large to small scales following the previous scheme. Next, as τ→ 2, Λ→+∞ and this means that the velocity gradient diverges in a finite time depending on Λ(0) and that contiguous particles trajectories diverge with a growth rate infinitely faster than velocity and temperature fields.

For ν> 0, χ> 0, then du/dt< 0 and dθ/dt< 0 in any case and *f* and $f_{\theta }$ are here supposed to be fully developed as soon as $d\lambda_{T}/dt$ = 0 and $d\lambda_{\theta }/dt$ = 0, respectively. These situations are qualitatively shown in the figure by the dashed lines for different values of $R_{T}$ and Pe, where $R_{T}$ = $\lambda_{T}u/\nu$ and Pe = $Pr\;R_{T}$ are, respectively, Reynolds number and Péclet number, both referred to the Taylor microscale, being Pr = ν/χ the Prandtl number. When the initial microscales are relatively large, the diffusivities effects are quite smaller than the convective terms, the energy cascade is initially stronger than the diffusivities effects and both the microscales exhibit about the same trend just discussed for ν = χ = 0. According to Equations (69) and (70), the interval where τ ranges can be splitted in two subregions for both *f* and $f_{\theta }$. The first ones correspond to values of τ∈(0,2) such that $d\lambda_{T}/dt<$ 0 and $d\lambda_{\theta }/dt<$ 0, which are upper bounded by the endpoints $\tau_{1}<$ 2, $\tau_{2}<$ 2 where $d\lambda_{T}/dt\left(\tau_{1}\right)$ = 0 and $d\lambda_{\theta }/dt\left(\tau_{2}\right)$ = 0 (dashed lines), respectively, being in general $\tau_{1}\neq \tau_{2}$. There, the kinetic and thermal energy cascades are momentarily balanced by viscosity and thermal diffusivity, respectively and both the autocorrelations can be considered fully developed. For both the correlations, such momentary balance happens in finite times τ< 2 which depend on the initial condition. As far as Λ is concerned, this initially coincides about with that obtained for ν = 0, then reaches its maximum for τ≲ 2 and thereafter diminishes due to viscosity. When Λ achieves its maximum, dΛ/dt = 0, chaos and mixing reach their maximum levels, the correlations are about fully developed, thus relative kinematics and fluid strain change much more rapidly than velocity field. Thereafter, we observe regions where dΛ/dt< 0. There, due to the relatively smaller values of the microscales, the dissipation is stronger than the energy cascade and both the correlation lengths tend to rise according to Equation (70). Such a region, which occurs immediately after the condition dΛ/dt = 0, corresponds to the regime of decaying turbulence.

Observe that the proposed closures (65) are expected to be verified where dΛ/dt> 0, in which the Navier–Stokes bifurcations generate the regime of fully developed turbulence. On the contrary, in regime of decaying turbulence –dΛ/dt< 0–, after a certain time, say $\tau^{+}>\tau_{1}\approx 2$, it results Λ/Λ(0)<1. In such situations, the relative kinematic and fluid strain could be not faster than velocity field, thus the statistical independence hypothesis (41) could be not satisfied and Equation (65) will be not defined. Therefore, the condition τ≈2 or Λ/Λ(0)<1 provides a further limit of validity for the proposed closure formulas.

## 12. *Self–Similarity and Developed Correlations of the Proposed Closures

This section analyzes self–similarity and developed shape of *f* and $f_{\theta }$ produced by the proposed closures. The new result with respect to the previous works consists in to remark that the proposed closures generate correlations self–similarity in proper ranges of *r*, which is directly related to the fluid trajectories divergence. To study this question, observe that a given function of *t* and *r*, say ψ=ψ(t,r), which completely exhibits self–similarity with respect to *r* as *t* changes, is a function of the kind

(74) $$\psi \left(t,r\right)=\psi \left(\frac{r}{\hat{L}\left(t\right)}\right)$$

and exactly satisfies the equation

(75) $$\begin{array}{c} \begin{array}{c} \frac{\partial \psi }{\partial t}=-\frac{\partial \psi }{\partial r}\frac{r}{\hat{L}}\frac{d\hat{L}}{dt}\equiv C\left(t\right)r\frac{\partial \psi }{\partial r},\\ C\left(t\right)=\frac{d\ln\hat{L}}{dt} \end{array} \end{array}$$

wherein $\hat{L}$ is the characteristic length associated with the specific problem. From such equation, the self–similarity of ψ is linked to the variation rate $d\ln\hat{L}\left(t\right)/dt$. Now, thanks to the mathematical structures of the proposed closures (65), and taking into account that *f* and $f_{\theta }$ are both even functions of *r* which near the origin behave like Equation (68), *K* and *G* can be expressed through even power series of *f* as follows

(76) $$\begin{array}{c} \begin{array}{c} K=u^{3}\sqrt{\frac{1-f}{2}}\frac{\partial f}{\partial r}=\frac{u^{3}}{2}\frac{r}{\lambda_{T}}\frac{\partial f}{\partial r}+\ldots =\frac{u^{2}}{2}\Lambda r\frac{\partial f}{\partial r}+\ldots \\ G=\theta^{2}u\sqrt{\frac{1-f}{2}}\frac{\partial f_{\theta }}{\partial r}=\frac{\theta^{2}u}{2}\frac{r}{\lambda_{T}}\frac{\partial f_{\theta }}{\partial r}+\ldots =\frac{\theta^{2}}{2}\Lambda r\frac{\partial f_{\theta }}{\partial r}+\ldots ,\\ \Lambda \propto \frac{d}{dt}{\langle \ln\varrho \rangle }_{\xi }\approx \left|\frac{d\ln\lambda_{T}}{dt}\right| \end{array} \end{array}$$

thus, the evolution equations of both the autocorrelations can be written in the following way

(77) $$\begin{array}{c} \begin{array}{c} \frac{\partial f}{\partial t}=u\sqrt{\frac{1-f}{2}}\frac{\partial f}{\partial r}+\ldots =\frac{u}{2\lambda_{T}}r\frac{\partial f}{\partial r}+\ldots =\frac{\Lambda }{2}r\frac{\partial f}{\partial r}+\ldots \\ \frac{\partial f_{\theta }}{\partial t}=u\sqrt{\frac{1-f}{2}}\frac{\partial f_{\theta }}{\partial r}+\ldots =\frac{u}{2\lambda_{T}}r\frac{\partial f_{\theta }}{\partial r}+\ldots =\frac{\Lambda }{2}r\frac{\partial f_{\theta }}{\partial r}+\ldots ,\\ \Lambda \propto \frac{d}{dt}{\langle \ln\varrho \rangle }_{\xi }\approx \left|\frac{d\ln\lambda_{T}}{dt}\right| \end{array} \end{array}$$

Comparing Equations (75) and (77), it follows that the proposed closures (65) generate self–similarity in a range of variation of *r* where Λ/2r∂f/∂r and $\Lambda /2r\partial f_{\theta }/\partial r$ are dominant with respect to the other terms. As the result, such self–similarity is directly caused by the continuous fluid trajectory divergence—quantified by Λ—which happens thank to very frequent kinematic bifurcations. In such these intervals, the correlations will exhibit self–similarity during their time evolution, thus *f* and $f_{\theta }$ can be expressed there as follows

(78) $$\begin{array}{c} \begin{array}{c} f\left(t,r\right)≃f\left(\frac{r}{\lambda_{T}\left(t\right)}\right),\\ f_{\theta }\left(t,r\right)≃f_{\theta }\left(\frac{r}{\lambda_{T}\left(t\right)}\right), \end{array} \end{array}$$

In such regions, the energy cascade is intensive and much stronger than the diffusivities effects, thus following Equation (70), $\lambda_{\theta }\left(t\right)$ is expected to be proportional to $\lambda_{T}\left(t\right)$

(79) $$\frac{\lambda_{\theta }\left(t\right)}{\lambda_{\theta }\left(0\right)}≃\frac{\lambda_{T}\left(t\right)}{\lambda_{T}\left(0\right)},$$

Next, as ϑ is a passive scalar, energy cascade and fluid diffusivities act on *u* and θ in such a way that their increments are proportional with each other. Therefore, far from the initial condition, we expect that

(80) $$\frac{\theta (t)}{\theta (0)}≃\frac{u(t)}{u(0)},$$

Now, Equation (79) provides a link between the correlation scales and Pr. In fact, substituting Equation (79) in Equation (69), we obtain

(81) $$\frac{\lambda_{\theta }}{\lambda_{T}}=\sqrt{\frac{6}{5}\frac{1}{Pr}}$$

Furthermore, from Equation (70), also $f^{IV}\left(0\right)$ and $f_{\theta }^{IV}\left(0\right)$ are related to the Prandtl number

(82) $$\frac{f_{\theta }^{IV}\left(0\right)}{f^{IV}\left(0\right)}=\frac{7}{3}Pr^{2}$$

Hence, the developed autocorrelations can be estimated searching for the solutions of the closed von Kármán–Howarth and Corrsin equations in the self–similar form (78) when $d\lambda_{T}/dt$ = $d\lambda_{\theta }/dt$ = 0. This leads to the following ordinary differential equations system

(83) $$\begin{array}{c} \begin{array}{c} \sqrt{\frac{1-f}{2}}\frac{df}{d\hat{r}}+\frac{2}{R_{T}}\left(\frac{d^{2}f}{d{\hat{r}}^{2}}+\frac{4}{\hat{r}}\frac{df}{d\hat{r}}\right)+\frac{10}{R_{T}}f=0,\\ \sqrt{\frac{1-f}{2}}\frac{df_{\theta }}{d\hat{r}}+\frac{2}{R_{T}Pr}\left(\frac{d^{2}f_{\theta }}{d{\hat{r}}^{2}}+\frac{2}{\hat{r}}\frac{df_{\theta }}{d\hat{r}}\right)+\frac{12}{R_{T}Pr}{\left(\frac{\lambda_{T}}{\lambda_{\theta }}\right)}^{2}f_{\theta }=0,\\ \hat{r}=\frac{r}{\lambda_{T}}. \end{array} \end{array}$$

Several solutions of these equations were numerically obtained in [2,4], where the author shows that velocity and temperature correlations agree with the Kolmogorov law, with the theoretical arguments of Obukhov–Corrsin and Batchelor and with the numerical simulations and experiments known from the literature [19,35,36,37,38,39,40,41].

**Figure 5:**
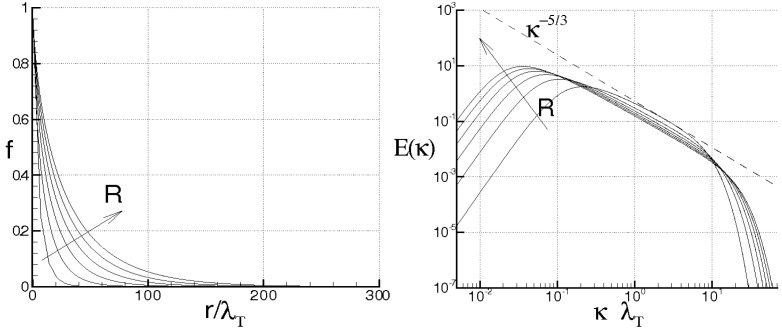
Longitudinal velocity correlations (**left**) and energy spectra (**right**) at different Taylor scale Reynolds numbers $R_{T}$ = 100, 200, 300, 400, 500, 600.

**Figure 6:**
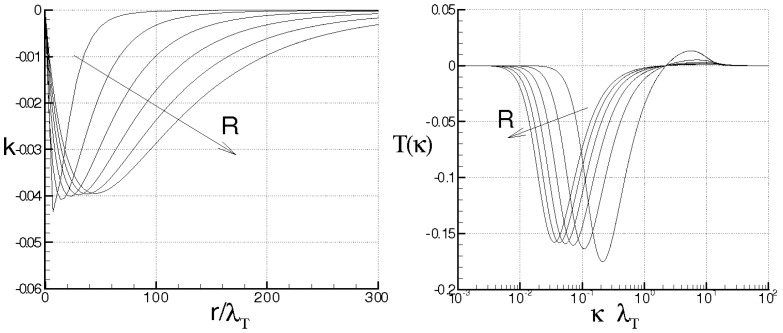
Triple longitudinal velocity correlations (**left**) and the corresponding spectra (**right**) at different Taylor scale Reynolds numbers $R_{T}$ = 100, 200, 300, 400, 500, 600.

For sake of reader convenience, Figure 5 and Figure 6 report the velocity correlations and the corresponding spectra E(κ), T(κ) numerically calculated with the first equation of Equation (83) for $R_{T}$ = 100, 200, 300, 400, 500, 600, being

(84) $$\left[\begin{array}{c} E(\kappa )\\ T(\kappa ) \end{array}\right]=\frac{1}{\pi }\int_{0}^{\infty }\left[\begin{array}{c} u^{2}f\left(r\right)\\ K(r) \end{array}\right]\kappa^{2}r^{2}\left(\frac{\sin\kappa r}{\kappa r}-\cos\kappa r\right)dr$$

where all these cases correspond to the same level of average kinetic energy. The integral correlation scale of *f* results to be a rising function of $R_{T}$, while the triple longitudinal velocity correlation *k* maintains negative with a minimum of about −0.04 whose value is achieved for values of $r/\lambda_{T}$ which rise with the Reynolds number. For what concerns the spectra, observe that increasing κ, the kinetic energy spectra behave like $E\left(\kappa \right)\approx \kappa^{4}$ near the origin, then exhibit a maximum and thereafter are about parallel to the dashed line $\kappa^{-5/3}$ in a given interval of the wave–numbers. The size of this latter, which defines the inertial range of Kolmogorov, rises as $R_{T}$ increases. For higher values of κ, which correspond to scales less than the Kolmogorov length, E(κ) decreases more rapidly than in the inertial range. As *K* does not modify the kinetic energy, the proposed closure gives $\int_{0}^{\infty }T\left(\kappa \right)d\kappa \equiv 0$.

From these solutions, the Kolmogorov constant *C*, here calculated as

(85) $$C=\max_{\kappa \in (0,\infty )}\frac{E\left(\kappa \right)\kappa^{5/3}}{\varepsilon^{2/3}}$$

is shown in Table 2 in function of the Reynolds number, where $\varepsilon =-3/2\;du^{2}/dt$. The obtained values of C≈2 are in good agreement with the corresponding values known from the literature.

**Figure 7:**
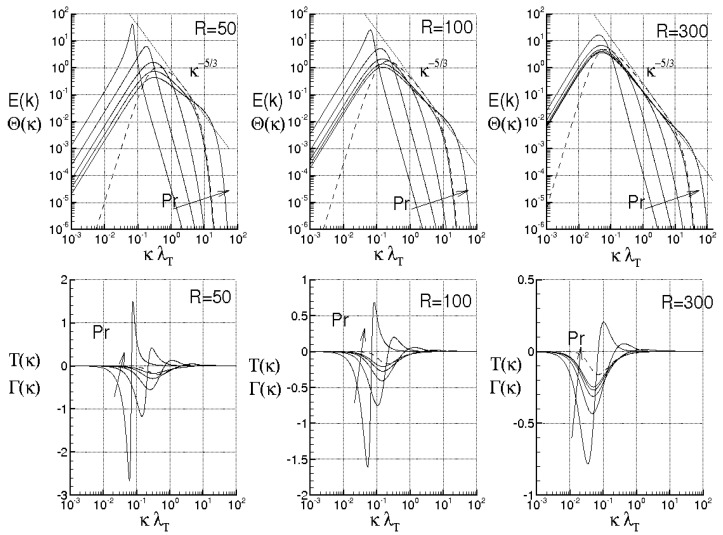
Spectra for Pr = 10^−3^, 10^−2^, 0.1, 1.0 and 10, at different Reynolds numbers. **Top**: kinetic energy spectrum E(κ) (dashed line) and temperature spectra Θ(κ) (solid lines). **Bottom**: velocity transfer function T(κ) (dashed line) and temperature transfer function Γ(κ) (solid line).

Next, Figure 7 shows the temperature spectra Θ(κ) and the temperature transfer function Γ(κ) calculated as follows [65]

(86) $$\begin{array}{c} \left[\begin{array}{c} \Theta (\kappa )\\ \Gamma (\kappa ) \end{array}\right]=\frac{2}{\pi }\int_{0}^{\infty }\left[\begin{array}{c} \theta^{2}f_{\theta }\left(r\right)\\ G(r) \end{array}\right]\kappa r\sin\kappa r\;dr \end{array}$$

in such a way that

(87) $$\int_{0}^{\infty }\Theta \left(\kappa \right)\;d\kappa =\theta^{2},\;\;\;\int_{0}^{\infty }\Gamma \left(\kappa \right)\;d\kappa =0$$

The variations of Θ(κ) with $R_{T}$ and Pr are quite peculiar and consistent with previous studies according to which there are regions where Θ(κ) exhibits different scaling laws $\Theta \left(\kappa \right)\approx \kappa^{n}$.

Following the proposed closures, n≃ 2 when κ→ 0 in any case. For Pr= 0.001, when $R_{T}$ ranges from 50 to 300, the temperature spectrum essentially exhibits two regions: one in proximity of the origin where n≃2 and the other one, at higher values of κ, where −17/3<n<−11/3, (value very close to −13/3). The value of n≈−13/3, here obtained in an interval around to $\hat{r}\approx$1, is in between the exponent proposed by [36] (−17/3) and the value determined by [40] (−11/3) by means of numerical simulations. Increasing κ, *n* significantly diminishes and Θ(κ) does not show scaling law. When Pr= 0.01, an interval near $\hat{r}\approx 1$ where −17/3<n<−13/3 appears and this is in agreement with [36]. Next, for Pr = 0.1, the previous scaling law vanishes, whereas for $R_{T}=$ 50 and 100, *n* changes with κ and Θ(κ) does not show clear scaling laws. When R=300, the birth of a small region is observed, where n≈−5/3 has an inflection point. For Pr= 0.7 and 1, with $R_{T}=$ 300, the width of this region is increased, whereas at Pr = 10 and R= 300, we observe two regions: one interval where *n* has a local minimum with n≃−5/3 and the other one where *n* exhibits a relative maximum, with n≃−1. For larger κ, *n* diminishes and the scaling laws disappear. The presence of the scaling law n≃−5/3 agrees with the theoretical arguments of [20,37] (see also [39,41] and references therein). Figure 7 also reports (on the bottom) the spectra Γ(κ) (solid lines) and T(κ) (dashed lines) which describe the energy cascade mechanism.

## 13. *Bifurcation Analysis of Closed von Kármán–Howarth Equation: From Fully Developed Turbulence Toward Non–Chaotic Regimes

Starting from non–chaotic regimes, the transition towards the fully developed turbulence happens through intermediate stages [34,42,43,51] which correspond to bifurcations where the relative Reynolds numbers show the same order of magnitude. This section presents a specific bifurcation analysis, which, unlike the classical route toward the chaos [34,42,43,51], analyzes the inverse route: the starting condition is represented by the fully developed homogeneous isotropic turbulence and the route followed is that towards the non–chaotic regime. Such route corresponds to the path B→A of Figure 1f,g and Figure 2. Along the line B→A, $R_{T}$ gradually diminishes and the bifurcations of the closed von Kármán–Howarth equation, properly defined, will be here studied. This analysis estimates $R_{T}^{*}$ through the closures (65) and their previously seen properties, where $R_{T}^{*}$ defines the minimum value of $R_{T}$ for which the turbulence maintains fully developed, homogeneous and isotropic. This provides the order of maginitude of Re at the transition, indicating a further limit of the proposed closures.

In order to formulate a bifurcation analysis for the velocity correlation equation, consider now the various coefficients of the closed von Kármán–Howarth equation which arise from the even Taylor series expansion of $f\left(t,r\right)=\sum_{k}f_{0}^{\left(k\right)}r^{k}/k!$. Each of such these coefficients corresponds to one of the following equations

(88) $$\left\{\begin{array}{c} \frac{du}{dt}=-5\nu \frac{u}{\lambda_{T}^{2}},\\ \frac{d\lambda_{T}}{dt}=-\frac{u}{2}+\frac{\nu }{\lambda_{T}}\left(\frac{7}{3}f_{0}^{IV}\lambda_{T}^{4}-5\right),\\ \frac{df_{0}^{IV}}{dt}=\ldots ,\\ \ldots \\ \frac{df_{0}^{\left(n\right)}}{dt}=\ldots ,\\ \ldots \end{array}\right.$$

Such equations can be written by introducing the infinite dimensional state vector

(89) $$Y\equiv \left(u,\lambda_{T},f_{0}^{IV},\ldots .f_{0}^{\left(n\right)},\ldots \right).$$

which represents the state of the longitudinal velocity correlation. Therefore, Equation (88), formally written as

(90) $$\dot{Y}=F\left(Y,\nu \right)$$

are equivalent to the closed von Kármán–Howarth equation. Equation (90) defines a bifurcation problem where ν plays the role of control parameter. Thus, this bifurcation analysis studies the variations of Y caused by ν according to

(91) $$F\left(Y,\nu \right)=F\left(Y_{0},\nu_{0}\right)$$

For $\nu >\nu_{0}$, Y is formally calculated through the implicit functions inversion theorem

(92) $$Y=G\left(Y_{0},\nu_{0},\nu \right)\equiv Y_{0}-\int_{\nu_{0}}^{\nu }{\left(\nabla_{Y}F\right)}^{-1}\frac{\partial F}{\partial \nu }\;d\nu$$

where $\nabla_{Y}F$ is the jacobian ∂F/∂y. A bifurcation of Equation (90) happens when this jacobian is singular, that is,

(93) $$\det\left(\nabla_{Y}F\right)=0$$

If $\nu_{0}$ is quite small ($R_{T}$ properly large), the energy cascade is dominant with respect to the viscosity effects and $\nabla_{Y}F$ is expected to be nonsingular. Increasing ν, Y smoothly varies according to Equation (92) and thereafter the dissipation gradually becomes stronger than the energy cascade until reaching the first bifurcation where condition (93) occurs. With reference to Figure 2, this corresponds to the path B→A until to reach *A*. There, a hard loss of stability is expected for the fully developed turbulence toward non–chaotic regimes [66]. Therefore, $R_{T}^{*}$ is calculated as that value of $R_{T}$ at bifurcation which gives the maximum of the largest real part of the eigenvalues of $\nabla_{Y}F$ [66,67] compatible with the current value of the average kinetic energy $u^{2}$, that is,

(94) $$\begin{array}{c} \begin{array}{c} R_{T}^{*}\;|\;\underset{k}{\sup}\left\{ℜ(l_{k})\right\}=\max,\\ \det\left(\nabla_{Y}F\right)=0,\\ u^{2}=\mathrm{given} \end{array} \end{array}$$

where $l_{k}$, *k*=1, 2,… are the eigenvalues of $\nabla_{Y}F$.

On the other hand, as previously seen, far from the initial condition, the energy cascade acts keeping *f* similar in the time in a given interval of variation of *r*. There, the evolution of *f* is expected to be described—at least in first approximation—by Equation (78) and this suggests that—under such approximation—the knowledge of *u* and $\lambda_{T}$ can be considered to be sufficient to describe the evolution of *f*. Hence, only the first two components of the state vector Y are taken which correspond to the coefficients of the order of $r^{0}$ and $r^{2}$ of Equation (88). Thus, thanks to the self–similarity, the infinite dimensional space where Y lies is replaced by a finite dimensional manifold and the state vector is reduced to

(95) $$Y\equiv \left(u,\lambda_{T}\right),$$

$f_{0}^{IV}$ plays the role of a parameter which characterizes the velocity correlation and the jacobian $\nabla_{Y}F$ reads as

(96) $$\begin{array}{c} \nabla_{Y}F=\left(\begin{array}{cc} \frac{\partial \dot{u}}{\partial u} & \frac{\partial \dot{u}}{\partial \lambda_{T}}\\ \frac{\partial \dot{\lambda_{T}}}{\partial u} & \frac{\partial \dot{\lambda_{T}}}{\partial \lambda_{T}} \end{array}\right) \end{array}$$

whose determinant is

(97) $$\det\left(\nabla_{Y}F\right)=-\frac{5\nu^{2}}{\lambda_{T}^{2}}\left(7f_{0}^{IV}\lambda_{T}^{2}+\frac{10}{\lambda_{T}^{2}}\right)+5\nu \frac{u}{\lambda_{T}^{3}}$$

From Equation (97), as long as ν>0 is properly small, $\det\left(\nabla_{Y}F\right)>0$. In order that a bifurcation happen, $\det\left(\nabla_{Y}F\right)$ must vanish for a certain value of ν and this implies that $f_{0}^{IV}\lambda_{T}^{4}>-10/7$. Thus, increasing ν, $\det\left(\nabla_{Y}F\right)/\nu$ diminishes and there exists a value of ν where this jacobian determinant vanishes. To determine $R_{T}^{*}$, $f_{0}^{IV}$ is eliminated through the bifurcation condition ($\det\left(\nabla_{Y}F\right)=0$) and Equation (97), that is,

(98) $$f_{0}^{IV}=\frac{1}{7\lambda_{T}^{2}\nu }\left(\frac{u}{\lambda_{T}}-10\frac{\nu }{\lambda_{T}^{2}}\right)$$

Therefore, the singular jacobian is

(99) $$\begin{array}{c} \nabla_{Y}F=\left(\begin{array}{cc} -5\nu /\lambda_{T}^{2} & 10\nu u/\lambda_{T}^{3}\\ -1/2 & u/\lambda_{T} \end{array}\right) \end{array}$$

and admits the following eigenvalues and eigenvectors $l_{1}$, $l_{2}$, $y_{1}$ and $y_{2}$, respectively

(100) $$\begin{array}{c} l_{1}=0,\;\;y_{1}=\left(u,\frac{\lambda_{T}}{2}\right)\\ l_{2}=\frac{u^{2}}{\nu }\left(\frac{1}{R_{T}}-\frac{5}{R_{T}^{2}}\right),\;\;y_{2}=\left(u,R_{T}\frac{\lambda_{T}}{10}\right) \end{array}$$

The eigenvalue $l_{2}\in R$ maintains positive for $R_{T}>5$ and reaches its maximum $l_{2max}=5\nu /\lambda_{T}^{2}$ for $R_{T}$ = 10. Accordingly, $R_{T}^{*}$ is estimated as

(101) $$R_{T}^{*}=10$$

which corresponds to $f_{0}^{IV}$=0.

Another characteristic value of $R_{T}$ is obtained in the case where both the eigenvalues vanish. This is $R_{T}$ = 5 and is expected to represent the onset of the decaying turbulence regime. In fact, in such situation, it is reasonable that *f* and $\lambda_{T}$ are

(102) $$\begin{array}{c} \frac{d\lambda_{T}}{dt}≃0,\\ f≃\exp\left(-\frac{1}{2}{\left(\frac{r}{\lambda_{T}}\right)}^{2}\right) \end{array}$$

Hence, $f_{0}^{IV}\lambda_{T}^{4}≃$3 and $R_{T}≃$ 4, in agreement with the previous estimation.

**Remark** **2.** It is worth remarking that $R_{T}^{*}$ provides the minimum of $R_{T}$ in fully developed isotropic homogeneous turbulence, thus this gives the order of magnitude of $R_{T}$ at the transition. Of course, the transition toward the chaos consists in intermediate stages (bifurcations of Navier–Stokes equations) where the turbulence is not developed and the velocity statistics does not exhibit, in general, isotropy and homogeneity. Hence, the obtained results provide the order of magnitude of $R_{T}$ at the transition. On the basis of this analysis, during the transition, $R_{T}$ ranges as

(103) $$4≲R_{T}≲10$$

The obtained value of $R_{T}^{*}$ = 10 is in very good agreement with the bifurcations analysis of the turbulent energy cascade [3], where the author shows that, in the transition toward the developed turbulence, if the bifurcations cascade follows the Feigenbaum scenario [42,43], the critical Taylor scale Reynolds number is about 10.13 and occurs after three bifurcations.

We conclude this section by remarking the limits under which $R_{T}^{*}$ is estimated. Such limits derive from the local self–similarity produced by the closures (65) which allow to consider only the first two equations of (88).

## 14. Velocity and Temperature Fluctuations

The purpose of this section is to obtain, by means of the previous Lyapunov analysis, formal expressions of velocity and temperature fluctuations which will be useful for estimating the statistics of these latter. For sake of our convenience, Navier–Stokes and thermal energy equations are now written in the following dimensionless divergence form

(104) $$\begin{array}{c} \frac{\partial u}{\partial t}=\mathrm{div}\;\hat{T},\\ \frac{\partial \vartheta }{\partial t}=-\mathrm{div}\;\hat{q} \end{array}\;\;\;\mathrm{in}\;\mathrm{which}\;\;\;\begin{array}{c} \hat{T}=T-u⊗u,\\ \hat{q}=q+u\vartheta \end{array}$$

where T and q denote, respectively, dimensionless stress tensor and heat flux, according to the Navier-Fourier laws

(105) $$\begin{array}{c} \begin{array}{c} T=-Ip+T_{v},\\ T_{v}=\frac{1}{Re}\left(\nabla_{x}u+\nabla_{x}u^{T}\right),\\ q=-\frac{1}{Pe}\nabla_{x}\vartheta \end{array} \end{array}$$

being I the identity tensor, $T_{v}$ the viscous stress tensor and the pressure *p* is given according to Equation (6).

In order to obtain the analytical forms of velocity and temperature fluctuations, Equation (104) are first expressed in terms of referential coordinate $x_{0}$

(106) $$\begin{array}{c} \frac{\partial u_{i}}{\partial t}=\left(\frac{\partial {\hat{T}}_{ij}}{\partial x_{0k}}\right)\left(\frac{\partial x_{0k}}{\partial x_{j}}\right)\equiv \left(\frac{\partial {\hat{T}}_{ij}}{\partial x_{0k}}\right)\;G_{jk}^{-1}\exp\left(-\tilde{\Lambda }t\right),\;\;i=1,2,3\\ \frac{\partial \vartheta }{\partial t}=-\left(\frac{\partial {\hat{q}}_{j}}{\partial x_{0k}}\right)\left(\frac{\partial x_{0k}}{\partial x_{j}}\right)\equiv -\left(\frac{\partial {\hat{q}}_{j}}{\partial x_{0k}}\right)\;G_{jk}^{-1}\exp\left(-\tilde{\Lambda }t\right) \end{array}$$

where the repeated index denotes the summation convention. The adoption of the referential coordinates allows to factorize of ∂u/∂t and ∂ϑ/∂t as a product of two statistically uncorrelated matrices: one depending on velocity and temperature fields and the other representing the local fluid deformation. Velocity and temperature fluctuations are here obtained integrating Equation (106) in the set (t,a). Due to the alignment property of the Lyapunov vectors [56], $\exp(-\tilde{\Lambda }t)$ rapidly goes to zero as t→∞ in any case, whereas $\partial {\hat{T}}_{ij}/\partial x_{0k}$ and $\partial {\hat{q}}_{j}/\partial x_{0k}$ are functions of slow growth of *t*. Hence, velocity and temperature fluctuations are formally calculated integrating Equation (106) in the set (t,∞) where $\partial {\hat{T}}_{ij}/\partial x_{0k}$ and $\partial {\hat{q}}_{j}/\partial x_{0k}$ are considered to be constant and equal to the corresponding values at the current time. Such fluctuations are then expressed in function of current velocity and temperature fields according to

(107) $$\begin{array}{c} u_{i}=\left(\frac{\partial {\hat{T}}_{ij}}{\partial x_{0k}}\right)\;W_{jk},\;\;i=1,2,3\\ \vartheta =-\left(\frac{\partial {\hat{q}}_{j}}{\partial x_{0k}}\right)\;W_{jk} \end{array}$$

being

(108) $$W_{jk}=\int_{0}^{\infty }G_{jk}^{-1}\exp\left(-\tilde{\Lambda }t\right)\;dt$$

where $|W_{jk}|<\infty$ as $G_{jk}^{-1}$ is represented by slow growth functions of *t*.

It is worth to remark that Equation (107) are, in general, rough approximations of velocity and temperature fluctuations. Nevertheless, in fully developed turbulence, dx(t) is considered to be much more rapid than u(t,x), thus Equation (107) provide one accurate way to express velocity and temperature in terms of referential coordinates by means of the Lyapunov theory.

## 15. *Statistics of Velocity and Temperature Difference

In developed turbulence, longitudinal velocity and temperature difference, $\Delta u_{r}$ = $(u\left(t,x^{\prime }\right)-u\left(t,x\right))\cdot r/r$ and Δϑ = $\vartheta \left(t,x^{\prime }\right)-\vartheta \left(t,x\right)$, $r=x^{\prime }-x$, play a role of paramount importance as these quantities describe energy cascade, intermittency and are linked to dissipation. This section analyzes the statistics of such quantities in fully developed homogeneous isotropic turbulence through the previously seen kinematic Lyapunov analysis and using a proper statistical decomposition of velocity and temperature. In order to determine this statistics, the Navier–Stokes bifurcations effect on $\Delta u_{r}$ and Δϑ is first analyzed. To this purpose, $\Delta u_{r}$ and Δϑ are expressed in function of current velocity and temperature through Equation (107)

(109) $$\begin{array}{c} \begin{array}{c} \Delta u_{r}={\left(\frac{\partial {\hat{T}}_{ij}}{\partial x_{0k}}\right)}^{\prime }\;W_{jk}^{\prime }-\left(\frac{\partial {\hat{T}}_{ij}}{\partial x_{0k}}\right)\;W_{jk}\\ \Delta \vartheta =-{\left(\frac{\partial {\hat{q}}_{j}}{\partial x_{0k}}\right)}^{\prime }\;W_{jk}^{\prime }+\left(\frac{\partial {\hat{q}}_{j}}{\partial x_{0k}}\right)\;W_{jk} \end{array} \end{array}$$

The several bifurcations happening during the fluid motion determine a continuous doubling of u in several functions, say ${\hat{v}}_{k}$, *k* = 1, 2, …, in the sense that each encountered bifurcation introduces new functions ${\hat{v}}_{k}$ whose characteristics are independent of the velocity field at previous time. Then, due to bifurcations, u is of the form

(110) $$u\left(t,x\right)\approx \sum_{k}{\hat{v}}_{k}\left(t,x\right),$$

It is worth remarking that, while u(t,x) is solution of the Navier–Stokes equations, the functions ${\hat{v}}_{k}$ are not. Therefore, the functions ${\hat{v}}_{k}$ are the result of the mathematical segregation due to bifurcations of a fluid state variable which physically only exist in combination, thus each of them is not directly observable. This implies that u will be distributed, in line with the Liouville theorem, according to a classical definite positive distribution function. On the contrary, each single function ${\hat{v}}_{k}$, representing mathematical segregation of the fluid state, will be distributed following extended distribution functions which can exhibit negative values [68,69,70] compatible with conditions linked to the specific problem. These conditions mainly arise from (a) the Navier–Stokes equations and from (b) the isotropic hypothesis. For what concerns (a), in order that pressure and inertia forces can cause sizable variations of velocity autocorrelation, each term ${\hat{v}}_{k}\equiv \left({\hat{v}}_{1},{\hat{v}}_{2},{\hat{v}}_{3}\right)$ will be distributed following highly nonsymmetric extended distribution function, for which

(111) $$\frac{|\langle {\hat{v}}_{ki}^{3}\rangle |}{{\langle {\hat{v}}_{ki}^{2}\rangle }^{3/2}}>>>1,\;\;i=1,2,3$$

As for (b), due to isotropic hypothesis, u would be distributed following a gaussian PDF [18], thus, according to the Navier–Stokes equations, pressure and inertia forces will not give contribution to the time derivative of the third statistical moment of u. Accordingly, the absolute value of odd statistical moments of order n of ${\hat{v}}_{k}$ is expected to be very high in comparison with the even statistical moments of order n + 1, that is,

(112) $$\frac{|\langle {\hat{v}}_{ki}^{n}\rangle |}{{\langle {\hat{v}}_{ki}^{2}\rangle }^{n/2}}>>>\frac{|\langle {\hat{v}}_{ki}^{n+1}\rangle |}{{\langle {\hat{v}}_{ki}^{2}\rangle }^{(n+1)/2}},\;\;n=3,5,7,\ldots ,\;\;\;i=1,2,3.$$

This suggests that Δu and u can be expressed through a specific statistical decomposition [71], as a linear combination of opportune stochastic variables $\xi_{k}$ that reproduce the doubling bifurcations effect and whose extended distribution functions satisfy Equations (111) and (112). Furthermore, as ϑ is a passive scalar, its fluctuations are the result of u and of thermal diffusivity, thus also ϑ is written by means of the same decomposition

(113) $$\begin{array}{c} u=\sum_{k}U_{k}\xi_{k},\\ \vartheta =\sum_{k}\Theta_{k}\xi_{k} \end{array}$$

where $U_{k}$ and $\Theta_{k}$(*k* = 1, 2, …) are coordinate functions of *t* and x, being $\nabla_{x}\cdot U_{k}=0,\;\forall k$ and $\xi_{k}$ (*k* = 1, 2, …) are dimensionless independent centered stochastic variables such that

(114) $$\langle \xi_{k}\rangle =0,\;\;\langle \xi_{i}\xi_{j}\rangle =\delta_{ij},\;\;\langle \xi_{i}\xi_{j}\xi_{k}\rangle =\left\{\begin{array}{c} q\neq 0,\;\forall \;i=j=k\\ 0\;\mathrm{else} \end{array}\right.$$

where *q*, providing the skewness of $\xi_{k}$ k = 1, 2…, satisfies to

(115) $$|q|>>>1,\;\langle \xi_{i}^{2}\rangle ,\;\langle \xi_{i}^{4}\rangle ,\;i=1,2,\ldots ,$$

Therefore, the distribution functions of $\xi_{k}$ can assume negative values compatible with Equations (114) and (115).

Through the decomposition (113), we will show that the negative value of $H_{u}^{\left(3\right)}\left(r\right)$ has very important implications for what concerns the statistics of $\Delta u_{r}$ and Δϑ, with particular reference to the intermittency of these latter which rises as Reynolds number and Péclet number increase. To study this question, consider first the analytical forms of the fluctuations of $u_{i}$ and ϑ in terms of $\xi_{k}$ obtained by substituting Equation (113) into Equation (107)

(116) $$\begin{array}{c} \begin{array}{c} u_{i}=\sum_{j}\sum_{k}A_{jk}^{\left(i\right)}\xi_{j}\xi_{k}+\frac{1}{R_{T}}\sum_{k}a_{k}^{\left(i\right)}\xi_{k},\;i=1,2,3\\ \vartheta =\sum_{j}\sum_{k}B_{jk}\xi_{j}\xi_{k}+\frac{1}{Pe}\sum_{k}b_{k}\xi_{k}, \end{array} \end{array}$$

where $\sum_{j}\sum_{k}A_{jk}^{\left(i\right)}\xi_{j}\xi_{k}$ and $1/R_{T}\sum_{k}a_{k}^{\left(i\right)}\xi_{k}$ are the contributions of inertia and pressure forces and of the fluid viscosity, respectively, whereas $\sum_{j}\sum_{k}B_{jk}\xi_{j}\xi_{k}$ and $1/Pe\sum_{k}b_{k}\xi_{k}$ arise from the convective term and fluid conduction. Because of turbulent isotropy, it is reasonable that $u_{i}$ and ϑ are both Gaussian stochastic variables [18,71,72], thus the various terms of Equation (116) satisfy the Lindeberg condition, a very general, necessary and sufficient condition for satisfying the central limit theorem [71,72]. Such theorem does not apply to $\Delta u_{i}$ and Δϑ as these latter are the difference between two correlated Gaussian variables, thus their PDF are expected to be very different with respect to Gaussian distributions. To study the statistics of $\Delta u_{r}$ and Δϑ, the fluctuations of these latter are first expressed in terms of $\xi_{k}$

(117) $$\begin{array}{c} \begin{array}{c} \Delta u_{r}\left(r\right)=\sum_{j}\sum_{k}\Delta A_{jk}\xi_{j}\xi_{k}+\frac{1}{R_{T}}\sum_{k}\Delta a_{k}\xi_{k},\\ \Delta \vartheta \left(r\right)=\sum_{j}\sum_{k}\Delta B_{jk}\xi_{j}\xi_{k}+\frac{1}{Pe}\sum_{k}\Delta b_{k}\xi_{k}, \end{array} \end{array}$$

being

(118) $$\begin{array}{c} \begin{array}{c} \Delta A_{jk}=\sum_{i=1}^{3}\left(A_{jk}^{\left(i\right)}\left(x+r\right)-A_{jk}^{\left(i\right)}\left(x\right)\right)\frac{r_{i}}{r}\equiv S_{ujk}+\Omega_{ujk},\\ \Delta a_{k}=\sum_{i=1}^{3}\left(a_{k}^{\left(i\right)}\left(x+r\right)-a_{k}^{\left(i\right)}\left(x\right)\right)\frac{r_{i}}{r},\\ \Delta B_{jk}=B_{jk}\left(x+r\right)-B_{jk}\left(x\right)\equiv S_{\theta jk}+\Omega_{\theta jk},\\ \Delta b_{k}=b_{k}\left(x+r\right)-b_{k}\left(x\right), \end{array} \end{array}$$

In Equation (118), the matrices $\Delta A_{jk}$ and $\Delta B_{jk}$ are decomposed following their symmetric and antisymmetric parts, respectively $S_{ujk}$, $S_{\theta jk}$ and $\Omega_{ujk}$, $\Omega_{\theta jk}$. These last ones give null contribution in Equation (117), whereas the terms arising from $S_{ujk}$ and $S_{\theta jk}$ are expressed as

(119) $$\begin{array}{c} \begin{array}{c} \sum_{j}\sum_{k}S_{Xjk}\xi_{j}\xi_{k}=\sum_{i}S_{Xii}\xi_{i}^{2}+\sum_{j\neq k}S_{Xjk}\xi_{j}\xi_{k},\\ X=u,\theta \end{array} \end{array}$$

in which the first term of Equation (119) is decomposed in the following manner

(120) $$\begin{array}{c} \begin{array}{c} \begin{array}{c} \sum_{i}S_{Xii}\xi_{i}^{2}=S_{X}^{+}\left(\eta_{X}^{2}-\sum_{j\neq k}^{+}\xi_{j}\xi_{k}\right)+\sum_{i}^{+}\left(S_{Xii}-S_{X}^{+}\right)\xi_{i}^{2}+S_{X}^{-}\left(\zeta_{X}^{2}-\sum_{j\neq k}^{-}\xi_{j}\xi_{k}\right)+\sum_{i}^{-}\left(S_{Xii}-S_{X}^{-}\right)\xi_{i}^{2},\\ X=u,\theta \end{array} \end{array} \end{array}$$

being

(121) $$\begin{array}{c} \begin{array}{c} \eta_{X}=\sum_{i}^{+}\xi_{i},\\ \zeta_{X}=\sum_{j}^{-}\xi_{j},\\ S_{X}^{+}=\frac{1}{n_{X}^{+}}\sum_{i}^{+}S_{ii}>0,\\ S_{X}^{-}=\frac{1}{n_{X}^{-}}\sum_{i}^{-}S_{ii}<0,\\ X=u,\theta , \end{array} \end{array}$$

and

(122) $$\begin{array}{c} \begin{array}{c} \begin{array}{c} \xi_{X}=-S_{X}^{+}\sum_{j\neq k}^{+}\xi_{j}\xi_{k}+\sum_{i}^{+}\left(S_{Xii}-S_{X}^{+}\right)\xi_{i}^{2}-S_{X}^{-}\sum_{j\neq k}^{-}\xi_{j}\xi_{k}+\sum_{i}^{-}\left(S_{Xii}-S_{X}^{-}\right)\xi_{i}^{2}+\sum_{j\neq k}S_{Xjk}\xi_{j}\xi_{k}+\sum_{k}\Delta a_{Xk}\xi_{k}\\ \equiv \sum_{ij}M_{Xij}\xi_{i}\xi_{j}+\sum_{k}g_{Xk}\xi_{k},\\ g_{uk}=\frac{\Delta a_{k}}{R_{T}},\;\;g_{\theta k}=\frac{\Delta b_{k}}{Pe},\;\;k=1,2,\ldots \\ X=u,\theta , \end{array} \end{array} \end{array}$$

where $\sum^{+}$ and $\sum^{-}$ denote summations for $\left(S_{Xjj}>0,S_{Xkk}>0\right)$ and $\left(S_{Xjj}\leq 0,S_{Xkk}\leq 0\right)$ and $n_{X}^{+}$ and $n_{X}^{-}$ are the corresponding numbers of terms of such summations, whereas $\sum_{j\neq k}^{+}$ and $\sum_{j\neq k}^{-}$ indicate the sums of addends calculated for j≠k corresponding to $S_{Xjj}>0$, $S_{Xkk}>0$ and $S_{Xjj}<0$, $S_{Xkk}<0$, respectively. The decomposition (119) and (120) and the definitions (121) lead to the following expression of velocity and temperature difference fluctuations

(123) $$\begin{array}{c} \begin{array}{c} \Delta u_{r}=\xi_{u}+S_{u}^{+}\eta_{u}^{2}+S_{u}^{-}\zeta_{u}^{2},\\ \Delta \vartheta =\xi_{\theta }+S_{\theta }^{+}\eta_{\theta }^{2}+S_{\theta }^{-}\zeta_{\theta }^{2}, \end{array} \end{array}$$

Now, we show that $\xi_{X}$, $\eta_{X}$ and $\zeta_{X}$, X=u,θ tend to uncorrelated gaussian variables. In fact, from Equation (121), $\eta_{X}$ and $\zeta_{X}$, X=u,θ are sums of random terms belonging to two different sets of uncorrelated stochastic variables (i.e., the sets for which $S_{Xii}<0$ and $S_{Xii}>0$), therefore $\eta_{X}$ and $\zeta_{X}$, are two uncorrelated stochastic variables such that $\langle \eta_{X}\rangle$ = $\langle \zeta_{X}\rangle$ = 0, *X* = u,θ. Furthermore, as $\xi_{k}$ are statistically independent with each other, the central limit theorem applied to Equation (121) guarantees that both $\eta_{X}$ and $\zeta_{X}$ tend to two uncorrelated centered gaussian random variables. As for $\xi_{X}$, *X* = u,θ, the following should be considered: due to the analytical structure of Equation (122), each term of $\xi_{X}$ is a centered variable, thus $\langle \xi_{X}\rangle$ = 0. Next, in Equation (122), the following terms $-S^{+}\sum_{j\neq k}^{+}\xi_{j}\xi_{k}+\sum_{i}^{+}\left(S_{Xii}-S_{X}^{+}\right)\xi_{i}^{2}$ and $-S_{X}^{-}\sum_{j\neq k}^{-}\xi_{j}\xi_{k}+\sum_{i}^{-}\left(S_{Xii}-S_{X}^{-}\right)\xi_{i}^{2}$ are mutually uncorrelated, as each of these is sum of random variables belonging to two different uncorrelated sets. Moreover, $\sum_{i\neq j}\xi_{i}\xi_{j}$ includes several weakly correlated terms, whereas $\sum_{k}g_{Xk}\xi_{k}$ is the sum of independent variables. On the other hand, due to hypothesis of fully developed chaos, the energy cascade, here represented by Equations (114), (115) and (117), will generate a strong mixing on the several terms of Equation (117), thus a proper variant of the central limit theorem can be applied to $\xi_{X}$ whose several terms are weakly dependent with each other [72]. As the result, $\xi_{X}$, *X* = u,θ will tend to centered gaussian variables statistically independent of $\eta_{X}$ and $\zeta_{X}$.

Hence, the statistics of $\Delta u_{r}$ and Δϑ is represented by the following structure functions of the independent centered gaussian stochastic variables $\xi_{X}$, $\eta_{X}$ and $\zeta_{X}$ for which $\langle \xi_{X}^{2}\rangle$ = $\langle \eta_{X}^{2}\rangle$ = $\langle \zeta_{X}^{2}\rangle$ = 1.

(124) $$\begin{array}{c} \begin{array}{c} \Delta u_{r}=L_{u}\xi_{u}+S_{u}^{+}\left(\eta_{u}^{2}-1\right)-S_{u}^{-}\left(\zeta_{u}^{2}-1\right),\\ \Delta \vartheta =L_{\theta }\xi_{\theta }+S_{\theta }^{+}\left(\eta_{\theta }^{2}-1\right)-S_{\theta }^{-}\left(\zeta_{\theta }^{2}-1\right), \end{array} \end{array}$$

where $L_{u}$ and $L_{\theta }$ are now introduced to take into account that $\xi_{X}$, $\eta_{X}$ and $\zeta_{X}$ have standard deviation equal to unity. Thus

(125) $$\begin{array}{c} \begin{array}{c} L_{u}\xi_{u}=\sum_{ij}M_{uij}\xi_{i}\xi_{j}+\frac{1}{R_{T}}\sum_{k}\Delta a_{uk}\xi_{k},\\ L_{\theta }\xi_{\theta }=\sum_{ij}M_{\theta ij}\xi_{i}\xi_{j}+\frac{1}{Pe}\sum_{k}\Delta a_{\theta k}\xi_{k}, \end{array} \end{array}$$

and $L_{X}$, $S_{X}^{-}$ and $S_{X}^{+}$ are parameters depending upon *r* which have to be determined. To this regard, it worth remarking that, in regime of fully developed isotropic turbulence in infinite domain, the numbers of parameters necessary to describe the statistics of $\Delta u_{r}$ and Δϑ should be minimum compatible with assigned quantities which define the current state of fluid motion, such as average kinetic energy, temperature standard deviation and correlation functions. On the other hand, the evolution equation of *f* [17] requires the knowledge of the correlations of the third order *k* to be solved. Therefore, in fully developed homogeneous isotropic turbulence, the sole knowledge of *f* and *k* is here considered to be the necessary and sufficient information for determining the statistics of $\Delta u_{r}$. This implies that $S_{u}^{+}$ is proportional to $S_{u}^{-}$ through a proper quantity which does not depend on *r*, that is,

(126) $$S_{u}^{+}\left(r\right)=\chi S_{u}^{-}\left(r\right)\equiv \chi S_{u}\left(r\right)$$

where χ < 1 is a function of $R_{T}$ giving the skewness of $\Delta u_{r}$, which has to be identified. Accordingly, $S_{u}$ and $L_{u}$ will be determined in function of *f* and *k* as soon as χ=χ(Re) is known. For what concerns the temperature difference, observe that, due to turbulence isotropy, the skewness of Δϑ should be equal to zero and this gives

(127) $$S_{\theta }^{+}\left(r\right)=S_{\theta }^{-}\left(r\right)\equiv S_{\theta }\left(r\right)$$

Therefore, the structure functions of $\Delta u_{r}$ and Δϑ read as

(128) $$\begin{array}{c} \begin{array}{c} \Delta u_{r}=L_{u}\xi_{u}+S_{u}\left(\chi \left(\eta_{u}^{2}-1\right)-\left(\zeta_{u}^{2}-1\right)\right),\\ \Delta \vartheta =L_{\theta }\xi_{\theta }+S_{\theta }\left(\eta_{\theta }^{2}-\zeta_{\theta }^{2}\right), \end{array} \end{array}$$

Furthermore, again following the parameters minimum number, the ratio $\Psi_{\theta }\left(r\right)\equiv S_{\theta }/L_{\theta }$ would be proportional to $\Psi_{u}\left(r\right)\equiv S_{u}/L_{u}$ through a proper coefficient depending upon the Prandtl number alone, that is

(129) $$\Psi_{\theta }\left(r\right)=\sigma \left(Pr\right)\Psi_{u}\left(r\right)$$

where σ is a function of the Prandtl number which has to be determined.

At this stage of the present analysis, we show that, in fully developed turbulence, $L_{u}$ and $L_{\theta }$ are, respectively, functions of $R_{T}$ and Pe, resulting in $L_{u}\propto R_{T}^{-1/2}$ and $L_{\theta }\propto Pe^{-1/2}$. In fact, from Equation (125) we obtain

(130) $$\begin{array}{c} \begin{array}{c} L_{u}^{2}=\sum_{ijkl}M_{uij}M_{ukl}\langle \xi_{i}\xi_{j}\xi_{k}\xi_{l}\rangle +\frac{2}{R_{T}}\sum_{k}M_{ukk}\Delta a_{uk}\langle \xi_{k}^{3}\rangle +\frac{1}{R_{T}^{2}}\sum_{k}\Delta a_{uk}^{2},\\ L_{\theta }^{2}=\sum_{ijkl}M_{\theta ij}M_{\theta kl}\langle \xi_{i}\xi_{j}\xi_{k}\xi_{l}\rangle +\frac{2}{Pe}\sum_{k}M_{\theta kk}\Delta a_{\theta k}\langle \xi_{k}^{3}\rangle +\frac{1}{Pe^{2}}\sum_{k}\Delta a_{\theta k}^{2}, \end{array} \end{array}$$

As $|\langle \xi_{k}^{3}\rangle |>>>1,\langle \xi_{i}\xi_{j}\xi_{k}\xi_{l}\rangle$, first and third addend of Equation (130) are negligible with respect to second one, thus $L_{u}$ and $L_{\theta }$ tend to functions of the kind

(131) $$\begin{array}{c} \begin{array}{c} L_{u}=\frac{F_{u}\left(r\right)}{\sqrt{R_{T}}},\\ L_{\theta }=\frac{F_{\theta }\left(r\right)}{\sqrt{Pe}}. \end{array} \end{array}$$

where $F_{u}\left(r\right)$ and $F_{\theta }\left(r\right)$ are functions of *r* which do not directly depend on $R_{T}$ and Pe. Hence, the dimensionless $\Delta u_{r}$ and Δϑ, normalized with respect to the corresponding standard deviations, are expressed in function of $R_{T}$ and Pe

(132) $$\begin{array}{c} \begin{array}{c} \frac{\Delta u_{r}}{\sqrt{\langle {\left(\Delta u_{r}\right)}^{2}\rangle }}=\frac{\xi_{u}+\Psi_{u}\left(\chi \left(\eta_{u}^{2}-1\right)-\left(\zeta_{u}^{2}-1\right)\right)}{\sqrt{1+2\Psi_{u}^{2}\left(1+\chi^{2}\right)}},\;\;\;\Psi_{u}\left(r\right)=\frac{S_{u}\left(r\right)}{L_{u}\left(r\right)}=\Phi \left(r\right)\sqrt{R_{T}},\\ \frac{\Delta \vartheta }{\sqrt{\langle {\left(\Delta \vartheta \right)}^{2}\rangle }}=\frac{\xi_{\theta }+\Psi_{\theta }\left(\eta_{\theta }^{2}-\zeta_{\theta }^{2}\right)}{\sqrt{1+4\Psi_{\theta }^{2}}},\;\;\;\Psi_{\theta }\left(r\right)=\frac{S_{\theta }\left(r\right)}{L_{\theta }\left(r\right)}=\Phi \left(r\right)\sqrt{Pe} \end{array} \end{array}$$

and this identifies $\sigma =\sqrt{Pr}$. Equation (132) provide peculiar structure functions giving the statistics of $\Delta u_{r}$ and Δϑ.

Now, if $\chi =\chi (R_{T})$ is considered to be known, $L_{u}$ and $S_{u}$ can be expressed in function of $\langle \Delta u_{r}^{2}\rangle$ and $\langle \Delta u_{r}^{3}\rangle$, where this latter is calculated adopting the proposed closure (65). In fact, $L_{u}$ and $S_{u}$ are related to $\langle \Delta u_{r}^{2}\rangle$ and $\langle \Delta u_{r}^{3}\rangle$ through Equation (128)

(133) $$\begin{array}{c} \begin{array}{c} \langle {\left(\Delta u_{r}\right)}^{3}\rangle =6u^{3}k=8S_{u}^{3}\left(\chi^{3}-1\right),\\ \langle {\left(\Delta u_{r}\right)}^{2}\rangle =2u^{2}\left(1-f\right)=L_{u}^{2}+2S_{u}^{2}\left(\chi^{2}+1\right), \end{array} \end{array}$$

thus, $L_{u}$, $S_{u}$ and Φ are expressed in function of f(r) and k(r) as

(134) $$\begin{array}{c} \begin{array}{c} S_{u}\left(r\right)={\left(\frac{3/4}{\chi^{3}-1}\right)}^{1/3}u\;k{\left(r\right)}^{1/3},\\ L_{u}\left(r\right)=\sqrt{2}\;u\sqrt{1-f\left(r\right)-\left(1+\chi^{2}\right){\left(\frac{3/4}{\chi^{3}-1}\right)}^{2/3}\;k{\left(r\right)}^{2/3}},\\ \Phi =\frac{S_{u}}{L_{u}}\frac{1}{\sqrt{R_{T}}} \end{array} \end{array}$$

In the expression of $L_{u}\left(r\right)$ of Equations (134), the argument of the square root must be greater than zero and this leads to the following implicit condition for χ

(135) $$\frac{1+\chi^{2}}{{\left(\chi^{3}-1\right)}^{2/3}}\leq \frac{1}{2}{\left(\frac{56}{3}\right)}^{2/3}$$

where the proposed closure (65) is taken into account. Inequality (135), solved with respect to χ, gives the upper limit for χ

(136) $$\chi \leq \chi_{\infty }=0.8659\ldots$$

As far as the temperature difference is concerned, we have

(137) $$\frac{\langle {\left(\Delta u_{r}\right)}^{2}\rangle }{\langle {\left(\Delta \vartheta \right)}^{2}\rangle }\equiv \frac{u^{2}}{\theta^{2}}\;\frac{1-f}{1-f_{\theta }}=\frac{L_{u}^{2}}{L_{\theta }^{2}}\;\frac{1+2\Psi_{u}^{2}\left(1+\chi^{2}\right)}{1+4\Psi_{\theta }^{2}}$$

thus Equation (137) allows to calculate $L_{\theta }$ in terms of the other quantities

(138) $$L_{\theta }=L_{u}\frac{\theta }{u}\sqrt{\frac{1-f_{\theta }}{1-f}}\sqrt{\frac{1+2\Phi^{2}R_{T}\left(1+\chi^{2}\right)}{1+4\Phi^{2}Pe}}$$

In Equations (134) and (138), the function χ = $\chi (R_{T})$ has to be identified, and Φ(r) depends on the specific shape of f(r), where, due to the constancy of $H_{u}^{\left(3\right)}\left(0\right)$, Φ(0) is assumed to be constant, independent of $R_{T}$.

The distribution functions of $\Delta u_{r}$ and Δϑ are formally calculated through the Frobenius–Perron equation [57], taking into account that $\xi_{X}$, $\eta_{X}$ and $\zeta_{X}$ are independent identically distributed centered gaussian variables such that $\langle \xi_{X}^{2}\rangle$ = $\langle \eta_{X}^{2}\rangle$ = $\langle \zeta_{X}^{2}\rangle$ = 1, X=u,θ

(139) $$\begin{array}{c} \begin{array}{c} F_{u}\left(\Delta u_{r}^{\prime }\right)=\int_{\xi }\int_{\eta }\int_{\zeta }P\left(\xi ,\eta ,\zeta \right)\;\delta \left(\Delta u_{r}^{\prime }-\Delta u_{r}\left(\xi ,\eta ,\zeta \right)\right)\;d\xi \;d\eta \;d\zeta ,\\ F_{\theta }\left(\Delta \vartheta^{\prime }\right)=\int_{\xi }\int_{\eta }\int_{\zeta }P\left(\xi ,\eta ,\zeta \right)\;\delta \left(\Delta \vartheta^{\prime }-\Delta \vartheta \left(\xi ,\eta ,\zeta \right)\right)\;d\xi \;d\eta \;d\zeta , \end{array} \end{array}$$

where δ is the Dirac delta, P(ξ,η,ζ) is the 3D gaussian PDF

(140) $$P\left(\xi ,\eta ,\zeta \right)=\frac{1}{\sqrt{{\left(2\pi \right)}^{3}}}\exp\left(-\frac{\xi^{2}+\eta^{2}+\zeta^{2}}{2}\right),$$

and $\Delta u_{r}\left(\xi ,\eta ,\zeta \right))$ and ϑ(ξ,η,ζ)) are determined by Equation (132).

In other words, the statistics of $\Delta u_{r}$ and Δϑ can be inferred looking at the proposed statistical decomposition (113) which includes the bifurcations effects in isotropic turbulence. This is a non–Gaussian statistics, where the absolute value of the dimensionless statistical moments increases with $R_{T}$ and Pe. In detail, the dimensionless statistical moments of $\Delta u_{r}$ and Δϑ are easily calculated in function of χ, $\Psi_{u}$ and $\Psi_{\theta }$

(141) $$\begin{array}{l} H_{u}^{\left(n\right)}\equiv \frac{\langle {\left(\Delta u_{r}\right)}^{n}\rangle }{{\langle {\left(\Delta u_{r}\right)}^{2}\rangle }^{n/2}}=\frac{1}{{\left(1+2\left(1+\chi^{2}\right)\Psi_{u}^{2}\right)}^{n/2}}\sum_{k=0}^{n}\left(\begin{array}{c} n\\ k \end{array}\right)\Psi_{u}^{k}\langle \xi_{u}^{n-k}\rangle \langle {\left(\chi \left(\eta_{u}^{2}-1\right)-\left(\zeta_{u}^{2}-1\right)\right)}^{k}\rangle ,\\ H_{\theta }^{\left(n\right)}\equiv \frac{\langle {\left(\Delta \vartheta \right)}^{n}\rangle }{{\langle {\left(\Delta \vartheta \right)}^{2}\rangle }^{n/2}}=\frac{1}{{\left(1+4\Psi_{\theta }^{2}\right)}^{n/2}}\sum_{k=0}^{n}\left(\begin{array}{c} n\\ k \end{array}\right)\Psi_{\theta }^{k}\langle \xi_{\theta }^{n-k}\rangle \langle {\left(\eta_{\theta }^{2}-\zeta_{\theta }^{2}\right)}^{k}\rangle , \end{array}$$

where Φ(0) and $\chi =\chi (R_{T})$ have to be identified. To this end, we first analyze the statistics of $\partial u_{r}/\partial r$ which, following the proposed Lyapunov analysis, exhibits a constant skewness $H_{u}^{\left(3\right)}\left(0\right)$ = −3/7. Then, $H_{u}^{\left(3\right)}\left(r\right)$ is first obtained from Equation (141)

(142) $$H_{u}^{\left(3\right)}\left(r\right)=\frac{8\Psi_{u}^{3}\left(\chi^{3}-1\right)}{{\left(1+2\Psi_{u}^{2}\left(1+\chi^{2}\right)\right)}^{3/2}}$$

and $H_{u}^{\left(3\right)}\left(0\right)$ is calculated for r→0

(143) $$H_{u}^{\left(3\right)}\left(0\right)=\frac{8\Psi_{u}^{3}\left(0\right)\left(\chi^{3}-1\right)}{{\left(1+2\Psi_{u}^{2}\left(0\right)\left(1+\chi^{2}\right)\right)}^{3/2}}$$

Accordingly, $\chi =\chi (R_{T})$ is implicitly expressed in function of $\Phi \left(0\right)\sqrt{R_{T}}$. From Equation (143), $\chi =\chi (R_{T})$ is a monotonic rising function of $R_{T}$ which, for $H_{u}^{\left(3\right)}\left(0\right)$ = −3/7, admits limit

(144) $$\chi_{\infty }=\lim_{R_{T}\to \infty }\chi \left(R_{T}\right)=0.8659\ldots$$

resulting in $\chi (R_{T})<0$ for properly small values of $R_{T}$. On the other hand, in fully developed turbulence, the PDF of $\partial u_{r}/\partial r$ exhibits non gaussian behavior (i.e., non gaussian tails) for $\partial u_{r}/\partial r\to \pm \infty$, accordingly χ must be positive. Hence, the limit condition χ=0 is supposed to be achieved for $R_{T}=R_{T}^{*}$ = 10 which represents the minimum value of $R_{T}$ for which the turbulence is homogeneous isotropic. This allows to identify Φ(0) by means of Equation (143)

(145) $$\Phi \left(0\right)=\frac{1}{\sqrt{R_{T}^{*}}}\sqrt{\frac{{H_{u0}^{\left(3\right)}}^{2/3}}{4-2{H_{u0}^{\left(3\right)}}^{2/3}}}=0.1409\ldots$$

**Figure 8:**
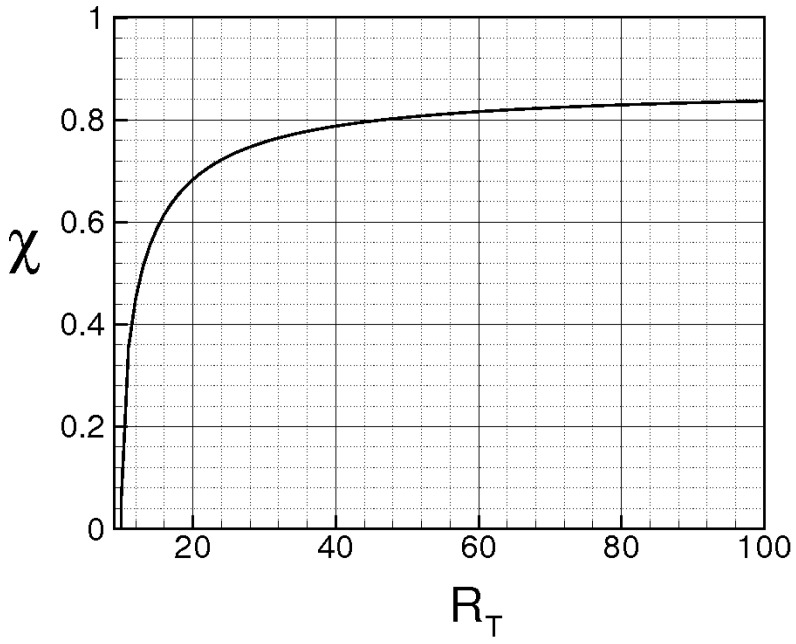
Characteristic Function χ = $\chi (R_{T})$.

Thus, Equation (143) gives, in the implicit form, the variation law $\chi =\chi (R_{T})$ which is depicted in Figure 8.

We conclude this section with the following considerations regarding the proposed analysis, and summarizing some of the results just obtained in the previous works.

For non–isotropic turbulence or in more complex situations with boundary conditions or walls, the velocity will be not distributed following a normal PDF, thus Equation (112) will be not verified, and Equation (132) will change its analytical structure incorporating stronger intermittent terms [72] giving the deviation with respect to the isotropic turbulence. Hence, the absolute statistical moments of $\Delta u_{r}$ will be greater than those calculated through Equation (141), indicating that, in more complex cases than the isotropic turbulence, the intermittency of $\Delta u_{r}$ can be significantly stronger.

Next, $\Psi_{u}$ and $\Psi_{\theta }$ represent the ratios (large scale velocity)–(small scale velocity) and (large scale temperature)–(small scale temperature), respectively. In particular, $\Psi_{u}\propto u/u_{s}\approx \left(u^{2}/\lambda_{T}\right)/\left(u_{s}^{2}/l_{s}\right)$ being $l_{s}$ and $u_{s}$ the characteristic small scale and the corresponding velocity. This means that $u/u_{s}\approx \lambda_{T}/l_{s}\approx \sqrt{R_{T}}$ and that the Reynolds number relative to $u_{s}$ and $l_{s}$ is $u_{s}l_{s}/\nu \approx$ 1, that is $l_{s}$ and $u_{s}$ identify the Kolmogorov scale and the corresponding velocity. For what concerns $\Psi_{\theta }$, ϑ is a passive scalar, thus $\Psi_{\theta }$ reads as $\Psi_{\theta }\propto \theta /\theta_{s}\approx \theta /\theta_{s}\left(u/\lambda_{T}\right)/\left(u_{s}/l_{s}\right)$ and this leads to $u_{s}l_{s}/\nu \approx$ 1.

At this stage of the present analysis, we can show that the kinematic bifurcation rate $S_{b}$, defined by Equation (25), is much larger than the kinematic Lyapunov exponents. In fact, $S_{b}$ can be also estimated as the ratio (large scale velocity)–(small scale length), where large scale velocity and small scale length are given by *u* and by the Kolmogorov scale, respectively. Taking into account the Kolmogorov scale definition and Equation (69), we obtain

(146) $$S_{b}\approx \frac{u}{l_{s}}=15^{1/4}R_{T}^{1/2}\Lambda$$

confirming the assumption made in the relative section. In fully developed turbulence, $S_{b}>>\Lambda$ and is a rising function of $R_{T}$.

As shown in Reference [1], the statistics given by Equations (139) and (141) agree with the experimental data presented in References [47,48]. There, in experiments using low temperature helium gas between two counter–rotating cylinders (closed cell), the PDF of $\partial u_{r}/\partial r$ and its statistical moments are measured. Although the experiments regard wall–bounded flows, the measured PDF of velocity difference are comparable with the present results (Equations (139) and (141)). Apart from a lightly non–monotonic evolution of $H_{u}^{\left(4\right)}\left(0\right)$ and $H_{u}^{\left(6\right)}\left(0\right)$ in [47,48], the dimensionless statistical moments of $\partial u_{r}/\partial r$ exhibit same trend and same order of magnitude of the corresponding quantities calculated with Equation (141). In particular, the PDFs of $\partial u_{r}/\partial r$ obtained with the present analysis show non gaussian tails which coincide with those measured in [47,48].

**Figure 9:**
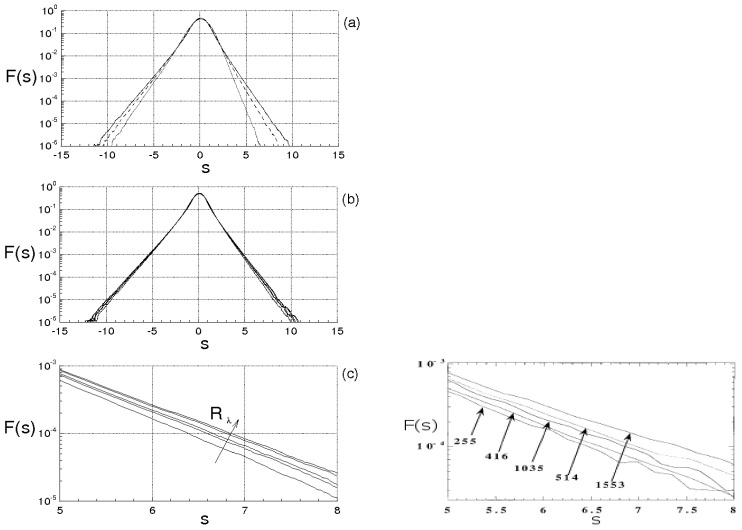
Left: PDF of $\partial u_{r}/\partial r$ for different values of $R_{T}$. (**a**) Dotted, dash–dotted and continuous lines are for $R_{T}$ = 15, 30 and 60, respectively. (**b**,**c**) PDFs for $R_{T}$ = 255, 416, 514, 1035 and 1553. (**c**) represents an enlarged part of the diagram (**b**). Right–bottom: Data from Reference [47].

In Figure 9, the normalized PDFs of $\partial u_{r}/\partial r$, calculated with Equations (139) and (141), are shown in terms of *s*

(147) $$s=\frac{\partial u_{r}/\partial r}{\sqrt{\langle {\left(\partial u_{r}/\partial r\right)}^{2}\rangle }}$$

in such a way that their standard deviations are equal to the unity. The results of Figure 9a are performed for $R_{T}$ = 15, 30 and 60, whereas Figure 9b,c report the PDF for $R_{T}$ = 255, 416, 514, 1035 and 1553, where Figure 9c represents the enlarged region of Figure 9b, showing the tails of PDF for 5<s<8. According to Equations (139) and (141), the tails of the PDF rise with the Reynolds number in the interval $10<R_{T}<700$, whereas for $R_{T}>700$, smaller variations are observed. On the right–bottom, the results of [47] for $R_{T}$ = 255, 416, 514, 1035 and 1553 are shown. Despite the aforementioned non–monotonic trend (see Figure 9 (Right–bottom)), Figure 9c gives values of the PDFs and of the corresponding average slopes which agree with those obtained in [47], expecially for 5<s<8. To this regards, it is worth to remark that, in certain conditions, the flow obtained in the experiments of [47] could be quite far from the isotropy hypothesis, as such experiments pertain wall–bounded flows, where the walls could significantly influence the fluid velocity in proximity of the probe.

**Figure 10:**
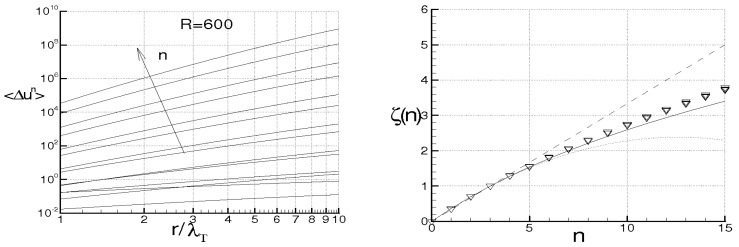
Left: Statistical moments of $u_{r}$ in terms of separation distance, for $R_{T}$ = 600. Right: Scaling exponents of $\partial u_{r}/\partial r$ at different $R_{T}$. Solid symbols are for the data calculated with the present analysis. Dashed line is for Kolmogorov K41 data [44]. Dotted line is for Kolmogorov K62 data [45]. Continuous line is for She–Leveque data [46].

In References [1,2,4] the scaling exponents $\zeta_{V}\left(n\right)$ associated with the several moments of $\Delta u_{r}$

(148) $$\langle {\left(\Delta u_{r}\right)}^{n}\rangle \approx A_{n}r^{\zeta_{V}\left(n\right)},$$

are calculated with Equation (132) through the following best fitting procedure. The statistical moments of $\Delta u_{r}$ are first calculated in function of *r* using Equation (141) (see Figure 10 (Left)). Then, the scaling exponents $\zeta_{V}\left(n\right)$ are identified through a minimum square method which, for each statistical moment, is applied to the following optimization problem

(149) $$J_{n}\left(\zeta_{V}\left(n\right),A_{n}\right)\;\equiv \int_{{\hat{r}}_{1}}^{{\hat{r}}_{2}}{\left(\langle {\left(\Delta u_{r}\right)}^{n}\rangle -A_{n}r^{\zeta_{V}\left(n\right)}\right)}^{2}dr=\min,\;n=1,2,\ldots$$

where $(\langle {\left(\Delta u_{r}\right)}^{n})\rangle$ are calculated with Equation (141), ${\hat{r}}_{1}$ is assumed to be equal to 0.1, whereas ${\hat{r}}_{2}$ is taken in such a way that $\zeta_{V}\left(3\right)$ = 1. The so obtained scaling exponents are shown in Figure 10 (Right–side) (solid symbols) where these are compared with those given by the Kolmogorov theories K41 [44] (dashed line) and K62 [45] (dotted line) and with the exponents calculated by She–Leveque [46] (continuous curve). For n<4, $\zeta_{V}\left(n\right)\approx n/3$ and for higher values of *n*, due to the nonlinear terms of Equation (132), $\zeta_{V}\left(n\right)$ shows multiscaling behavior. The values of $\zeta_{V}\left(n\right)$ here calculated are in good agreement with the She–Leveque data, and result to be lightly greater than those obtained in [46] for n> 8.

**Figure 11:**
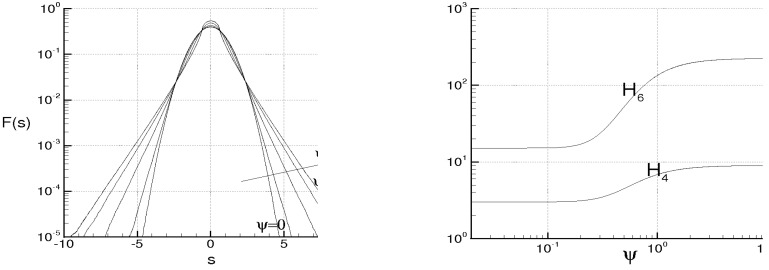
Left: Distribution function of the longitudinal temperature derivatives, at different values of $\Psi_{\theta }$. Right: Dimensionless statistical moments, $H_{\theta }^{\left(4\right)}$ and $H_{\theta }^{\left(6\right)}$ in function of $\Psi_{\theta }$.

As far as the temperature difference statistics is concerned, Figure 11 (Left) shows the distribution function of ∂ϑ/∂r in terms of dimensionless abscissa

(150) $$s=\frac{\partial \vartheta /\partial r}{\sqrt{\langle {\left(\partial \vartheta /\partial r\right)}^{2}\rangle }}$$

calculated with Equations (132) and (139), for different values of $\Psi_{\theta }$. To show the intermittency of such PDF, the flatness $H_{\theta }^{\left(4\right)}$ and the hyperflatness $H_{\theta }^{\left(6\right)}$, defined as

(151) $$H_{\theta }^{\left(4\right)}=\frac{\langle s^{4}\rangle }{{\langle s^{2}\rangle }^{2}},\;\;\;H_{\theta }^{\left(6\right)}=\frac{\langle s^{6}\rangle }{{\langle s^{2}\rangle }^{3}}$$

are plotted in Figure 11 (Right) in terms of $\Psi_{\theta }$. When $\Psi_{\theta }=$ 0, the PDF is gaussian, thus $H_{\theta }^{\left(4\right)}$ = 3 and $H_{\theta }^{\left(6\right)}$ = 15. Increasing $\Psi_{\theta }$, the non–linear terms $\eta_{\theta }$ and $\zeta_{\theta }$ cause an increment of $H_{\theta }^{\left(4\right)}$ and $H_{\theta }^{\left(6\right)}$ and when $\Psi_{\theta }\to \infty$ $H_{\theta }^{\left(4\right)}\to$ 9 and $H_{\theta }^{\left(6\right)}\to$ 225.

**Figure 12:**
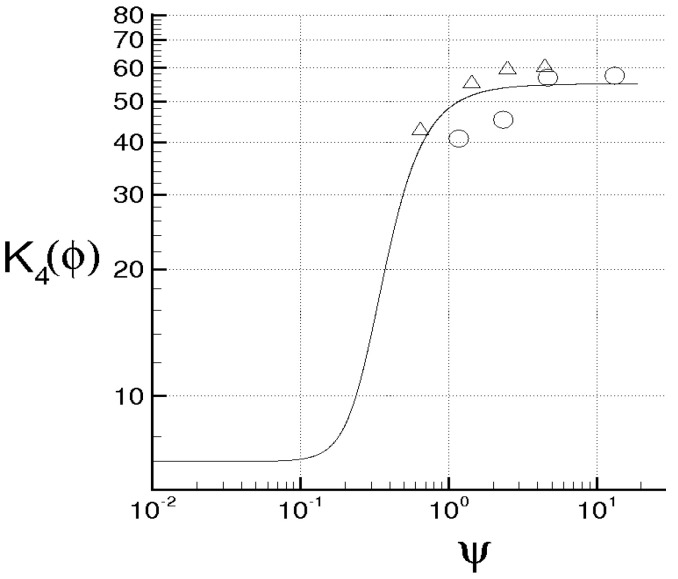
Comparison of the results: Kurtosis of temperature dissipation in function of $\Psi_{\theta }$. The symbols represent the results by [73].

Furthermore, the statistics of the temperature dissipation

(152) φ=χ∇ϑ·∇ϑ,

is analyzed in function of $\Psi_{\theta }$ with particular reference to its intermittency. To this end, the Kurtosis of φ, $K_{4}\left(\varphi \right)$, is estimated by means of Equation (141), where, thanks to isotropy, the three components of $\nabla \vartheta \equiv (\vartheta_{x},\vartheta_{y},\vartheta_{z})$ are identically distributed. Next, $\vartheta_{x}$, $\vartheta_{y}$ and $\vartheta_{z}$ are supposed to be statistically uncorrelated. This last assumption allows to estimate the Kurtosis of φ in terms of the dimensionless statistical moments of ∂ϑ/∂r, according to

(153) $$K_{4}\left(\varphi \right)=\frac{H_{\theta }^{\left(8\right)}-4H_{\theta }^{\left(6\right)}+6H_{\theta }^{\left(4\right)}-3}{3\left({\left(H_{\theta }^{\left(4\right)}\right)}^{2}+1-2H_{\theta }^{\left(4\right)}\right)}+2$$

where $H_{\theta }^{\left(4\right)}$, $H_{\theta }^{\left(6\right)}$ and $H_{\theta }^{\left(8\right)}$ are calculated using Equation (141). Figure 12 shows $K_{4}\left(\varphi \right)$ in function of $\Psi_{\theta }$, and compares the values calculated with the present theory (solid line), with those obtained by [73] through the nonlinear large–eddy simulations (symbols). The comparison shows that the data are in qualitatively good agreement. In more detail, for $\Psi_{\theta }\to \infty$, $K_{4}\to$ 55, whereas the results of Reference [73] give a value of around 60. This difference could be due to the fact that the present analysis only considers the isotropic turbulence which tends to bound the values of the dimensionless statistical moments of ∂ϑ/∂r and of φ and to the approximation of assuming the components of ∇ϑ to be statistically uncorrelated.

Finally, observe that the experimental data of [47,74] allow to identify Φ(0). Table 3 reports a comparison between the value of Φ(0) calculated with the present theory and those obtained through elaboration of the experimental data of [47,74]. Form this comparison, the value of Φ(0) calculated with Equation (145) is in very good agreement with those obtained through the elaboration the data of [47,74].

## 16. Conclusions

A review of previous theoretical results concerning an original turbulence theory is presented. The theoretical approaches here adopted, different with respect to the other articles, confirm and corroborate the results of the previous works.

In separate sections, novel issues regarding the proposed turbulence theory are presented, and are here summarized.

- The bifurcation rate of velocity gradient, calculated along fluid particles trajectories is shown to be much larger than the maximal Lyapunov exponent of the kinematic field.
- On the basis of the previous item, the energy cascade is viewed as a stretching and folding succession of fluid particles which gradually involves smaller and smaller scales.
- The central limit theorem, in the framework of the bifurcation analysis, provides reasonable argumentation that the finite time Lyapunov exponent can be approximated by a gaussian random variable if τ≈1/Λ.
- The closures of von Kármán–Howarth and Corrsin equations given by this theory determine velocity and temperature correlations which exhibit local self–similarity directly linked to the continuous particles trajectories divergence.
- The proposed bifurcation analysis of the closed von Kármán–Howarth equation studies the route from developed turbulence toward non–chaotic regimes and leads to an estimation of the critical Taylor scale Reynolds number in isotropic turbulence in agreement with the various experiments.
- Finally, a specific statistical decomposition of velocity and temperature is presented. This decomposition, adopting random variables distributed following extended distribution functions, leads to the statistics of velocity and temperature difference which agrees with the data of experiments.

## Figures and Tables

**Table 1:** Comparison of the results: Skewness of $\partial u_{r}/\partial r$ at diverse Taylor–scale Reynolds number $R_{T}\equiv u\lambda_{T}/\nu$ following different authors.

Reference	Simulation	$R_{T}$	$H_{3}\left(0\right)$
Present analysis	-	-	−3/7 = −0.428…
[59]	DNS	202	−0.44
[60]	DNS	45	−0.47
[61]	DNS	64	−0.40
[62]	LES	<71	−0.40
[63]	LES	∞	−0.40
[64]	LES	720	−0.42

**Table 2:** Kolmogorov constant for different Taylor-Scale Reynolds number.

$R_{T}$	C
100	1.8860
200	1.9451
300	1.9704
400	1.9847
500	1.9940
600	2.0005

**Table 3:** Identification of Φ(0) through elaboration of experimental data of [47,74] and comparison with the present analysis.

Reference	Φ(0)
Present Analysis	0.1409…
[47]	≃0.148
[74]	≃0.135
